# Bioactive Amino-Carbon Dots for Sustainable Crop Protection: Cellular Uptake and Metabolomic Insights into the Antifungal and Antibacterial Activity in Tomato Plants

**DOI:** 10.1021/acsami.6c03466

**Published:** 2026-04-30

**Authors:** Alessandro Camilli, Adriano Patriarca, Patrizia Ferrante, Elisa Brasili, Pierfrancesco Atanasio, Corrado Di Conzo, Elisa Sturabotti, Laura Verdolini, Simone D’Angeli, Marco Rossi, Fabrizio Vetica, Francesca Leonelli, Giovanna Simonetti, Alessio Valletta

**Affiliations:** 1 Department of Chemistry, 9311 Sapienza University of Rome, Piazzale Aldo Moro 5, 00185 Rome, Italy; 2 Consiglio per la Ricerca in Agricoltura e l’Analisi dell’Economia Agraria (CREA), Centro di Olivicoltura, Frutticoltura e Agrumicoltura (CREA-OFA), Via di Fioranello, 52, 00134 Roma, Italy; 3 Department of Environmental Biology, 9311 Sapienza University of Rome, Piazzale Aldo Moro 5, 00185 Rome, Italy; 4 NMR-Based Metabolomics Laboratory, University of Rome Sapienza, Piazzale Aldo Moro 5, 00185 Rome, Italy; 5 Department of Basic and Applied Sciences for Engineering (SBAI), 9311 Sapienza University of Rome, Via A. Scarpa 14, 00161 Rome, Italy; 6 Department of Applied Science and Technology (DISAT), Polytechnic of Turin, Corso Castelfilardo 39, 10129 Torino, Italy; 7 Center for Cooperative Research in Biomaterials (CIC biomaGUNE), Basque Research and Technology Alliance (BRTA), Donostia-San Sebastián 20014, Spain; 8 Department of Public Health and Infectious Diseases, 9311 Sapienza University of Rome, Piazzale Aldo Moro 5, 00185 Rome, Italy; 9 Research Centre for Nanotechnology Applied to Engineering of Sapienza University of Rome (CNIS), Piazzale A. Moro 5, 00185 Rome, Italy

**Keywords:** carbon dots, nanobiotechnology, integrated pest management, *Pseudomonas syringae* pv. tomato, *Botrytis cinerea*, metabolomics

## Abstract

Amino-functionalized carbon dots (CDs-NH_2_) are emerging as multifunctional nanomaterials for sustainable agriculture due to their tunable surface chemistry, water dispersibility, low toxicity, and inherent antimicrobial activity. In this study, we envisioned the application of CDs-NH_2_ as both antifungal and antibacterial materials against plant pathogens, thoroughly assessing CDs-NH_2_ internalization and their effects on plant growth and metabolomic profiles as well as defense responses to pathogen infection. Initially, CDs-NH_2_ were synthesized and fully characterized with a focus on morphology, structure, and their stability under biotic and abiotic environmental conditions. Antioxidant assays based on DPPH and ABTS radical scavenging demonstrated the redox activity of CDs-NH_2_. Fluorescence microscopy investigations demonstrated that CDs-NH_2_ can quickly penetrate plant and fungal cells. Confocal microscopy investigations, complemented by colocalization studies with endocytic tracer FM4-64, demonstrated that endocytosis is the primary mechanism for CDs-NH_2_ cellular uptake in *Botrytis cinerea*. Furthermore, CDs-NH_2_ were found to quickly penetrate plant cells, enhancing tomato seed germination and subsequent development. Quantitative chemical analyses indicated the absorption of CDs-NH_2_ via the root system of the rice seedlings. *In vitro* and *in planta* experiments have shown the efficacy of CDs-NH_2_ against phytopathogenic fungi (*Botrytis cinerea*) and bacteria (*Pseudomonas syringae* pv. *tomato*). *In vitro* antibacterial activity tests combined with metabolomic analyses via ^1^H NMR indicate that CDs-NH_2_ exert their antimicrobial activity straight on *P. syringae* and trigger defense responses in the plant upon infection. Overall, these findings highlight the dual role of CDs-NH_2_ as antivirulence agents and metabolic modulators, underscoring their potential as sustainable nanotools for integrated crop protection at the plant–pathogen interface while emphasizing the need for further investigation into their environmental and human safety.

## Graphical abstract

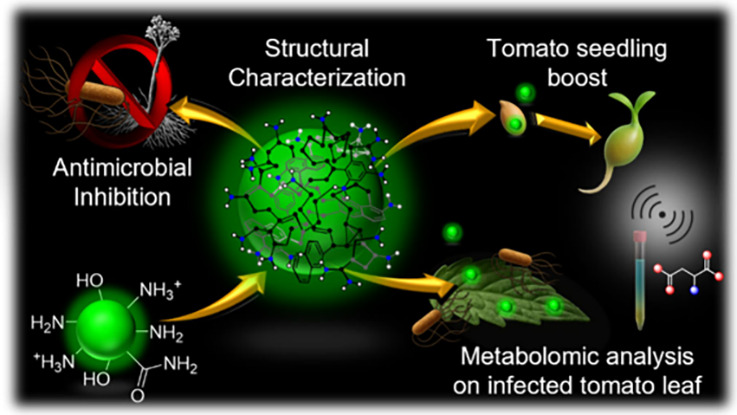

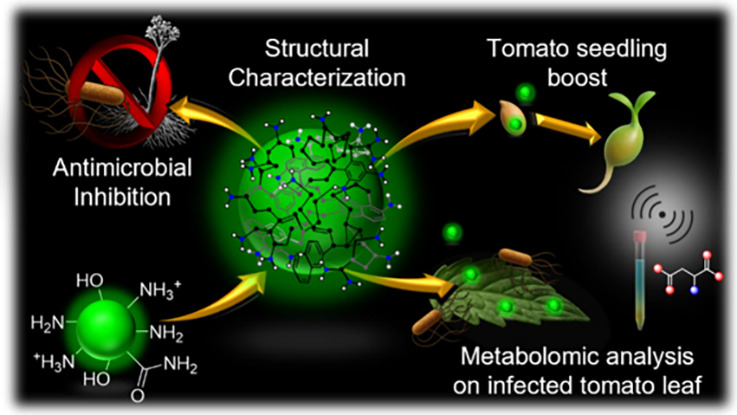

## 1 Introduction

Sustainable crop protection is a primary challenge for ensuring global food security, environmental sustainability, and public health.^1^ Tomato (*Solanum lycopersicum*), one of the most economically significant crops worldwide, is constantly threatened by pathogens such as the Gram-negative bacterium *Pseudomonas syringae* pv. *tomato*, responsible for bacterial specks, and the necrotrophic fungus *Botrytis cinerea*, the causal agent of gray mold.^2,3^ These pathogens can lead to yield losses of up to 75%, posing a major concern for Mediterranean economies that heavily depend on tomato cultivation.^4^ Traditionally, disease management has relied on intensive applications of conventional chemical plant protection products (PPPs), which have raised significant concerns regarding environmental pollution and the emergence of resistance.^5^ In this context, nanotechnology offers innovative solutions by providing nanomaterials capable of enhancing treatment efficiency and promoting crop growth with a reduced environmental footprint.^6,7^

Among the most promising nanomaterials are carbon dots (CDs), a novel class of self-fluorescent, zero-dimensional, and quasi-spherical nanoparticles (<10 nm) characterized by high biocompatibility.^8^ CDs can be synthesized from disparate precursors, such as small molecules, polymers, biomass, and biowastes via simple, economical, and sometimes eco-friendly methods based on solvothermal or dry heating at high temperatures.^9–12^ A key strategy to enhance their performance is nitrogen doping (N-CDs), as the presence of amino groups increases their solubility in aqueous media, modifies their electronic structure and fluorescence,^8,13–16^ and facilitates versatile chemical modifications, broadening their applicability to drug delivery,^17^ catalysis,^18–22^ sensing,^23^ and bioimaging.^24^ Additionally, N-CDs can act as nanocarriers for the targeted delivery of bioactive compounds, while simultaneously promoting photosynthetic processes and exhibiting intrinsic antimicrobial activity, thereby minimizing damage to the host plant.^25,26^

Although a broad range of biological and antimicrobial activities of N-CDs has been reported, key gaps remain in understanding their interactions with biological systems.^27^ However, recurring mechanisms can be identified, most of which are linked to their small size and surface functionalities.^28^ Although it has been observed that positively charged CDs interact with the membranes of human pathogens,^29^ the molecular pathways of cellular internalization and translocation within plant tissues and phytopathogenic fungi are not yet fully elucidated. Furthermore, literature lacks a systematic assessment of their environmental degradation, including standardized criteria and methodologies to evaluate their environmental and biological impacts.^30^ Indeed, some studies report benign byproducts, while others suggest the potential formation of toxic molecular species.^31,32^ In this scenario, the definition of the clear usefulness of CDs is hard to depict, and strong efforts are needed to harness the strength points that CDs have shown to possess in biochemistry. Therefore, a clear characterization of CDs is essential to rationalize (and ultimately clarify) the structure–activity relationship and the long-term safety of these nanomaterials, ultimately enabling their reliable application in precision agriculture.^33^

The innovation of the present study lies in a multidisciplinary approach combining advanced microscopy with metabolomics to investigate the behavior of CDs at the plant-pathogen interface. Building upon previous in vivo biocompatibility assessments,^29,34^ which demonstrated the safety of amino-decorated carbon dots (CDs-NH_2_) in both cellular and animal models, this work maps for the first time the mechanisms of endocytosis in *B. cinerea* and vascular translocation in plants such as *Oryza sativa*. The primary methodological novelty lies in the application of ^1^H-NMR-based metabolomics to quantify plant metabolite fluctuations. Additional efforts have been focused on the structural characterization of CDs-NH_2_ and the quantification of surface amino groups. Since the antimicrobial activity of N-CDs is already well-documented, we shifted our attention toward the ability of these nanomaterials to induce metabolic reprogramming (priming) in tomato plants. This approach aims to distinguish direct biocidal action from the plant’s physiological response to validate CDs-NH_2_ as biocompatible nanotools for truly sustainable crop protection.

## 2 Results and Discussion

### 2.1 Synthesis and Characterization of CDs-NH_2_

In this study, we aimed to present a class of amino-functionalized carbon dots (CDs-NH_2_), previously synthesized by our group, as a novel, effective, and sustainable nanoplatform for the control of crop phytopathogens. To this end, we optimized the purification procedure of our sample and carried out an in-depth structural and morphological characterization of CDs-NH_2_ to confirm their nanoscale features, shape, crystallinity, and surface functionality. Other characterizations can be found in our earlier publications.^29,34,35^ The main characterization results from those studies (NMR, FTIR, absorption, emission, elemental analysis, and XPS) have been included in the Supporting Information of the present work (Figures S2A–I).

Following centrifugation and filtration, an initial dialysis step was introduced using an aqueous 150 mM NaCl solution (followed by dialysis in sole water) to increase osmotic pressure and facilitate the removal of molecular byproducts that are difficult to eliminate by other methods.^36^ Prior to lyophilization, the CDs-NH_2_ solution was sonicated to prevent aggregation and ensure a more homogeneous drying process. This methodology was essential to obtain a material with a significantly higher volume, lower density, softer texture, and lighter weight with a color change from dark to light brown (Figure S1). The mass yield was approximately 16%, averaged at least over three reaction batches. Importantly, this new purification strategy did not alter the optical properties of CDs-NH_2_, including their characteristic fluorescence emission.^34^ Likewise, the surface functionalization remained unaffected, as confirmed by the high content of primary amines, estimated by the Kaiser test to be 1.22 ± 0.05 mmol of NH_2_/g of CDs-NH_2_ (eq 1, Figure S3). However, this test shows some limitations, since the formation of the adduct (Ruhemann’s purple) can be restricted by steric hindrance, by the not-fully complete reactivity of all primary amino groups, and by its limited stability.^37^ In addition, in our case, a further issue arose from the poor solubility of CDs-NH_2_ in the reaction mixture, which prevented an accurate quantification of the amino groups. For these reasons, we assessed the total primary amino groups through a more sensitive and accurate method based on the ^19^F NMR quantification of the imine groups following the functionalization of CDs-NH_2_ with a fluorinated aldehyde. To this end, we selected 4-fluorobenzaldehyde (FBA), since it is nonenolizable and the formation of imine functionalities leads to a change in the fluorine chemical shift due to direct conjugation with the para position. In this way, the signals of the functionalized CDs-NH_2_ can be separated from those of the fluorinated probe, as they appear in a distinct region of the ^19^F NMR spectrum. Therefore, we carried out the reaction between CDs-NH_2_ and FBA in DMSO-*d*_6_ at 25 °C for 24 h. The ^19^F NMR spectrum (Figures S4 and S5) revealed the presence of two sets of broad signals: one around –107 and –110 ppm, and the other around –114 and –115 ppm, compatible with the formation of iminium and imine functionalities, in agreement with what already reported in earlier works.^18,38^ The number of primary amino groups calculated using as standard the residual signal of the aldehyde was 1.90 mmol NH_2_/g CDs-NH_2_ (eq 2). As expected, this value was higher than the one estimated through the Kaiser test, thus proving to be a better and more accurate method for detecting and quantifying primary amino functionalities.

Diffusion-ordered spectroscopy (DOSY) experiments were performed to determine the homogeneity of the nanoparticles. In recent years, DOSY has emerged as a powerful technique for assessing sample purity.^36,39^ This aspect is particularly relevant to our objectives, considering that many of the properties commonly ascribed to CDs often belong to molecular side products, not adequately removed during the purification process. Therefore, the presence of well-aligned signals in the DOSY spectra serves as an indication that only a single species is present in the sample, diffusing with a unique diffusion coefficient. This evidence is a strong and pivotal indicator of CDs’ purity. In our case (Figure 1A), the DOSY spectrum of purified CDs-NH_2_ displayed very low polydispersity with an average diffusion coefficient of (1.55 ± 0.06) × 10^–6^ cm^2^/s (see Supporting Information for details), hence indicating good homogeneity of the nanoparticles.

**Figure 1:**
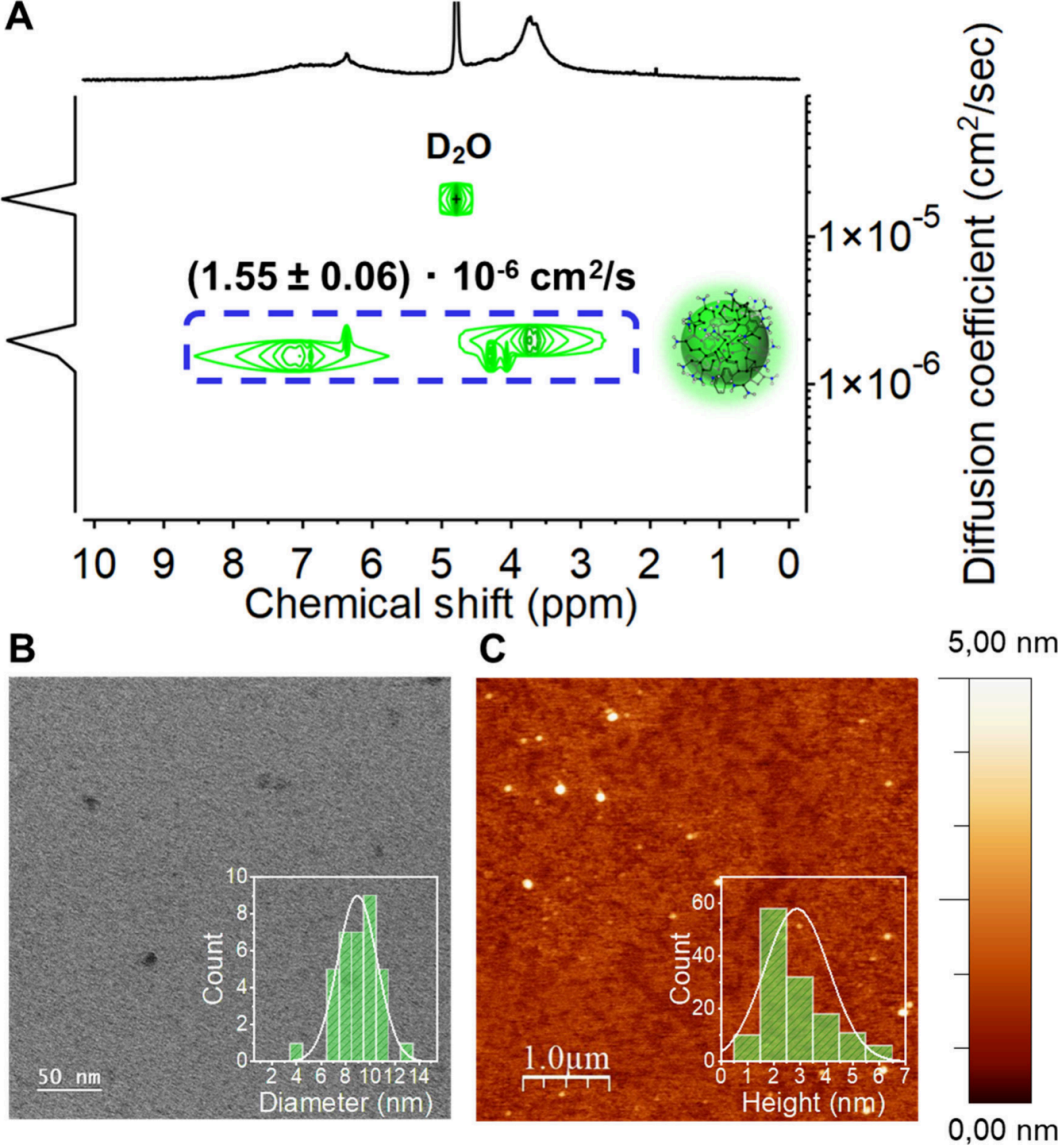
(A) DOSY spectrum (D_2_O, 400 MHz, 10 mg/mL) of CDs-NH_2_, calibrated against the residual water signal. Diffusion coefficients were determined to be (1.454 ± 0.100) × 10^–6^ cm^2^/s and (1.638 ± 0.044) × 10^–6^ cm^2^/s for aromatic and aliphatic protons, respectively. (B) TEM image and diameter distribution histogram of CDs-NH_2_, with lateral dimensions of 9.0 ± 1.6 nm. (C) AFM image and height distribution histogram with average particle size of 3.3 ± 1.4 nm.

Complementary TEM, AFM and STEM analyses revealed that CDs-NH_2_ consisted of nanoparticles with lateral dimensions of 9.0 ± 1.6 nm and a height of 3.3 ± 1.4 nm (Figures 1B,C, S7A–C, and S8), although some larger isolated or aggregated particles were also detected. These results collectively confirm that CDs-NH_2_ are quite homogeneous, quasi-spherical, and well-dispersed carbon nanoparticles, exhibiting high purity and structural consistency, in line with what is reported in literature for other types of CDs.^8,40^ These physicochemical features are particularly relevant for their intended application in plant and fungal systems, as not only small size but also particle uniformity, morphology, and dispersion state strongly influence their interaction with biological interfaces, thereby facilitating efficient penetration, cellular uptake, and transport within these organisms.^27,41^

Following the morphological characterization, energy-dispersive X-ray spectroscopy (EDS) was employed to gain insights into the elemental composition of CDs-NH_2_. The EDS spectrum, together with the corresponding elemental map showing the distribution of the Kα signals, confirmed the presence of carbon and nitrogen both within and on the surface of the nanoparticles (Figure S9A–C). However, due to the proximity of the carbon and nitrogen peak energies (C Kα at 276 eV and N Kα at 392 eV), nitrogen quantification was challenging, especially in the presence of a high carbon background. Nonetheless, the presence of nitrogen was supported by the elemental map (Figure S9C); moreover, the profile (Figure S9D) shows the presence of nitrogen along the entire diameter of the particle, a result further corroborated by the Kaiser test and ^19^F NMR analyses. Additional confirmation was obtained from previous elemental analysis (12.4% nitrogen), FTIR (3320 cm^–1^, N–H stretching), and XPS (402 and 400 eV) data reported in our earlier work^34^ and in Figure S2B,E,F,H. These results consistently confirm the presence of amino and ammonium groups on the CDs-NH_2_ surface.

Afterward, the structural organization of CDs-NH_2_ was investigated through a comparative analysis of electron diffraction, XRD, and Raman spectroscopy. The electron diffraction pattern of a single particle (Figure S10A,B) exhibits features typical of a polycrystalline material with distinct diffraction rings corresponding to the characteristic planes of graphite (the (111) ring appears broadened due to its proximity to the primary beam). Complementary XRD analysis of the bulk powder revealed a diffractogram with broad features typical of predominantly amorphous materials (Figure 2A). The absence of sharp peaks, including the (002) reflection of graphite at 2θ ≈ 26.5° (interlayer spacing of 3.35 Å), suggests a lack of long-range order in bulk materials, whereas single polycrystalline particles have been found using STEM analysis. These observations suggest that any graphitic domains are either too small, highly disordered, or localized to be detected by XRD. Overall, these data support the presence of short-range graphitic order confined within an overall disordered or amorphous carbon matrix, a structural motif commonly observed in carbon dots.^42^

**Figure 2:**
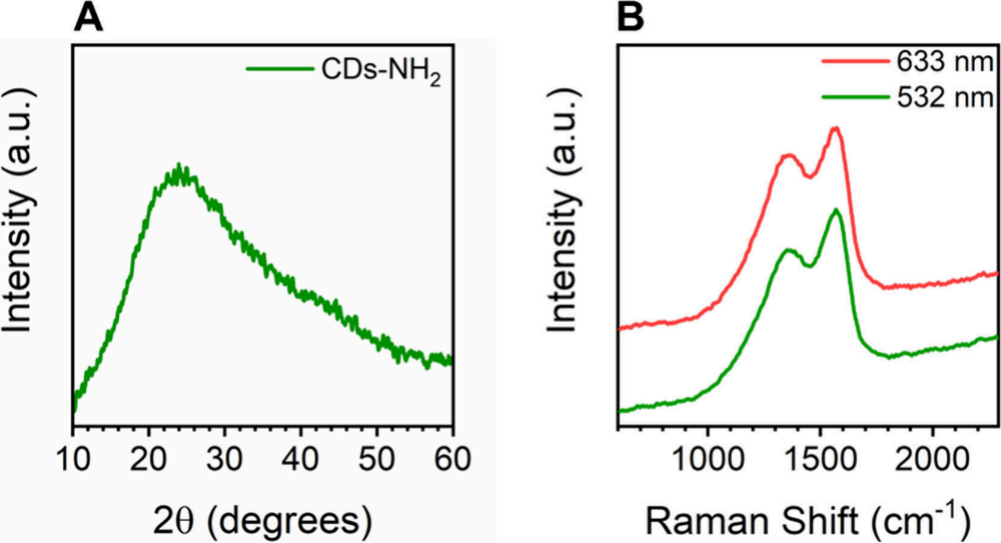
(A) X-ray diffractogram of CD-NH_2_ powder. (B) Raman spectra of CDs-NH_2_ obtained using 532 nm (green line) and 633 nm (red line) excitation wavelengths.

To complement the macroscopic structural insights from the diffraction results, Raman spectroscopy was employed for the localized analysis of CDs-NH_2_. Raman spectra acquired from various regions at 532 and 633 nm excitation wavelengths produced similar spectra (Figure 2B and Figure S11) and consistently showed the characteristic D (1354.1 ± 3.5 cm^–1^) and G (1594.9 ± 7.6 cm^–1^) bands of graphite, indicating the coexistence of graphitic domains and structural disorder. However, all spectra show a consistent amount of noise during measurement, presumably due to fluorescence, a highly defective structure, small particle size, and/or a limited amount of graphitic material within an amorphous matrix. Moreover, the presence of two additional bands, i.e., D_3_ (1551.1 ± 8.6 cm^–1^) and D_4_ (1206.3 ± 22.5 cm^–1^) bands, together with an *I*(D)/*I*(G) ratio of 0.79 ± 0.03, supports the presence of sp^3^-hybridized carbon atoms within a relatively disordered graphitic structure (Table S1, Figure S12).

Therefore, all these experimental observations support that CDs-NH_2_ are overall nearly spherical, green-fluorescent, and core–shell nanoparticles with dimensions below 10 nm, as revealed by microscopical analyses. Their internal structure features a hybrid carbonaceous core, in which regions of short-range graphitic ordering coexist with amorphous domains, suggesting partial crystallinity embedded within a largely disordered matrix. A surface shell enriched with amino groups, as confirmed by Kaiser test, ^19^F NMR and previous analyses, surrounds the internal core. Together with the inner core, superficial amines reasonably contribute to the dispersibility and stabilization of the dots in aqueous media,^43^ further mediating the interactions with the external biochemical environment. The presence of sp^3^-hybridized carbon atoms, evidenced by the additional D_3_ and D_4_ Raman bands, additionally corroborates the structural disorder and the high density of edge sites or defects. Due to their accessible amino functionalities, intrinsic green fluorescence, and small dimensions, CDs-NH_2_ may represent promising nanoprobes for bioimaging and antimicrobial applications. Furthermore, their safe-by-design nature and water solubility remark their suitability for biology.

### 2.2 Biointeraction and Uptake of CDs-NH_2_ in Fungal and Plant Models

Internalization in fungal and plant cells was investigated by confocal and light microscopy, exploiting the intrinsic green fluorescence of CDs-NH_2_. *B. cinerea* cells that were not incubated with CDs-NH_2_ (control) did not exhibit autofluorescence within the wavelength range used in this study. Following incubation in a CDs-NH_2_ suspension, an intense fluorescence signal was observed within the fungal cells (Figure 3). The fluorescence was initially localized in the cell walls (5–10 min after incubation) (Figure 3A,B) and then moved to the interior of the cells (10–40 min after incubation) (Figure 3C–F), where it was concentrated in bodies with the typical appearance of membrane vesicles (see Video S1 in Supporting Information). Compared to the extracellular environment, the cytoplasm showed a greater intensity of the fluorescent signal, indicating an active accumulation of CDs-NH_2_. A limited number of conidia showed fluorescence (Figure 3G,H).

**Figure 3:**
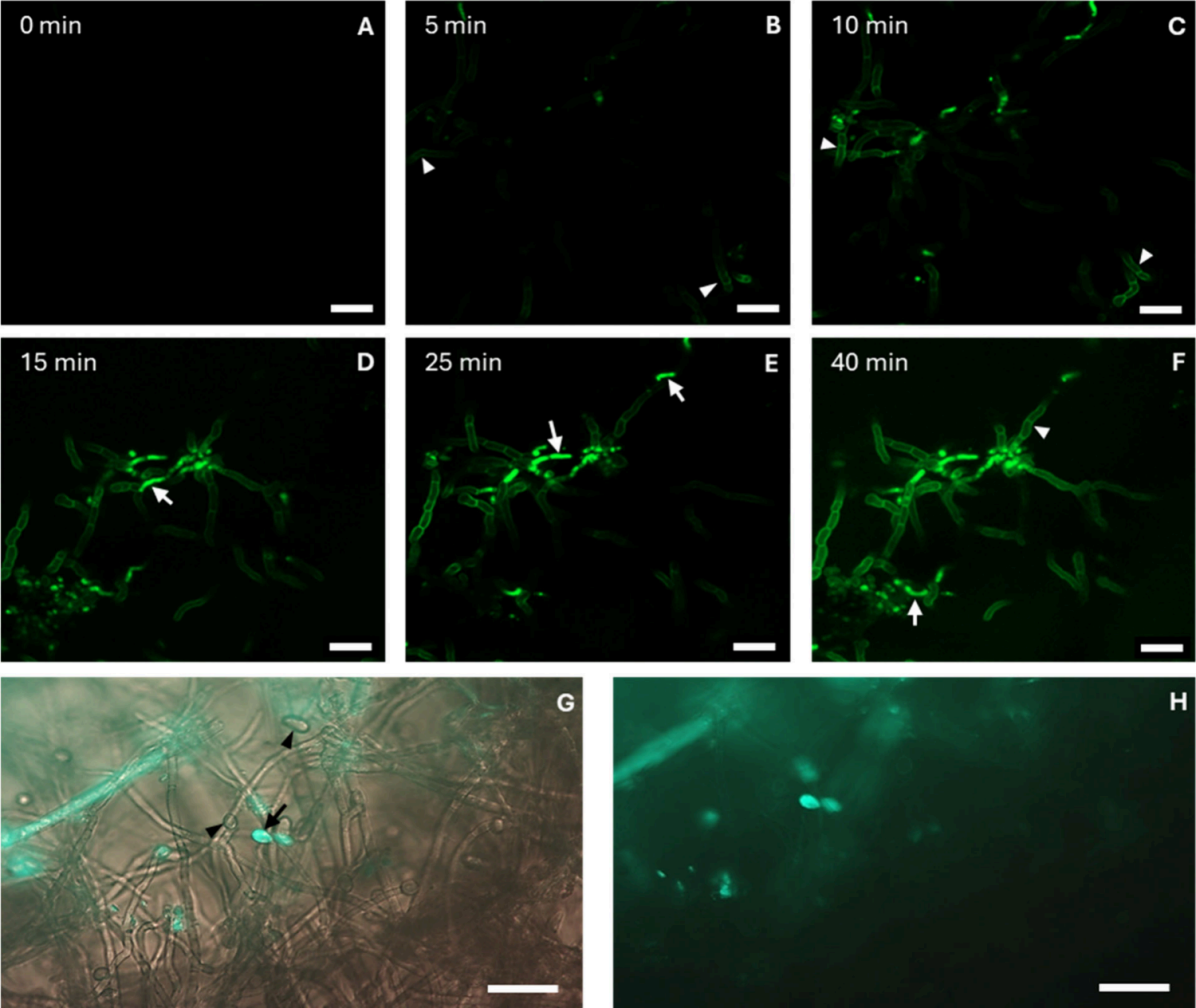
Cellular uptake of CDs-NH_2_ in *B. cinerea*. (A–F) Confocal microscopy images showing CDs-NH_2_ uptake at different time points postincubation; fluorescence is localized in the cell wall (white arrowheads) and within the cytoplasm of hyphal cells (white arrows). (G) Epifluorescence and (H) merged fluorescence/brightfield micrographs, demonstrating that some conidia exhibit fluorescence (black arrows) while others do not (black arrowheads). Scale bars: 20 μm.

Extant research suggests that the entry of carbon dots into the intracellular environment involves multiple pathways, modulated by intrinsic factors such as particle size, surface potential, and specific surface chemistry.^27,34^

To elucidate the mechanism by which carbon dots enter fungal cells, colocalization experiments between the FM4-64 probe and the nanoparticles were performed by using confocal microscopy (Figure 4). The high degree of colocalization, evidenced by Pearson’s correlation and Manders’ overlap coefficients, indicates that endocytosis is the primary uptake route for CDs-NH_2_ cellular internalization; however, the contribution of alternative mechanisms cannot be excluded. This observation is consistent with the currently available literature.^44^

**Figure 4:**
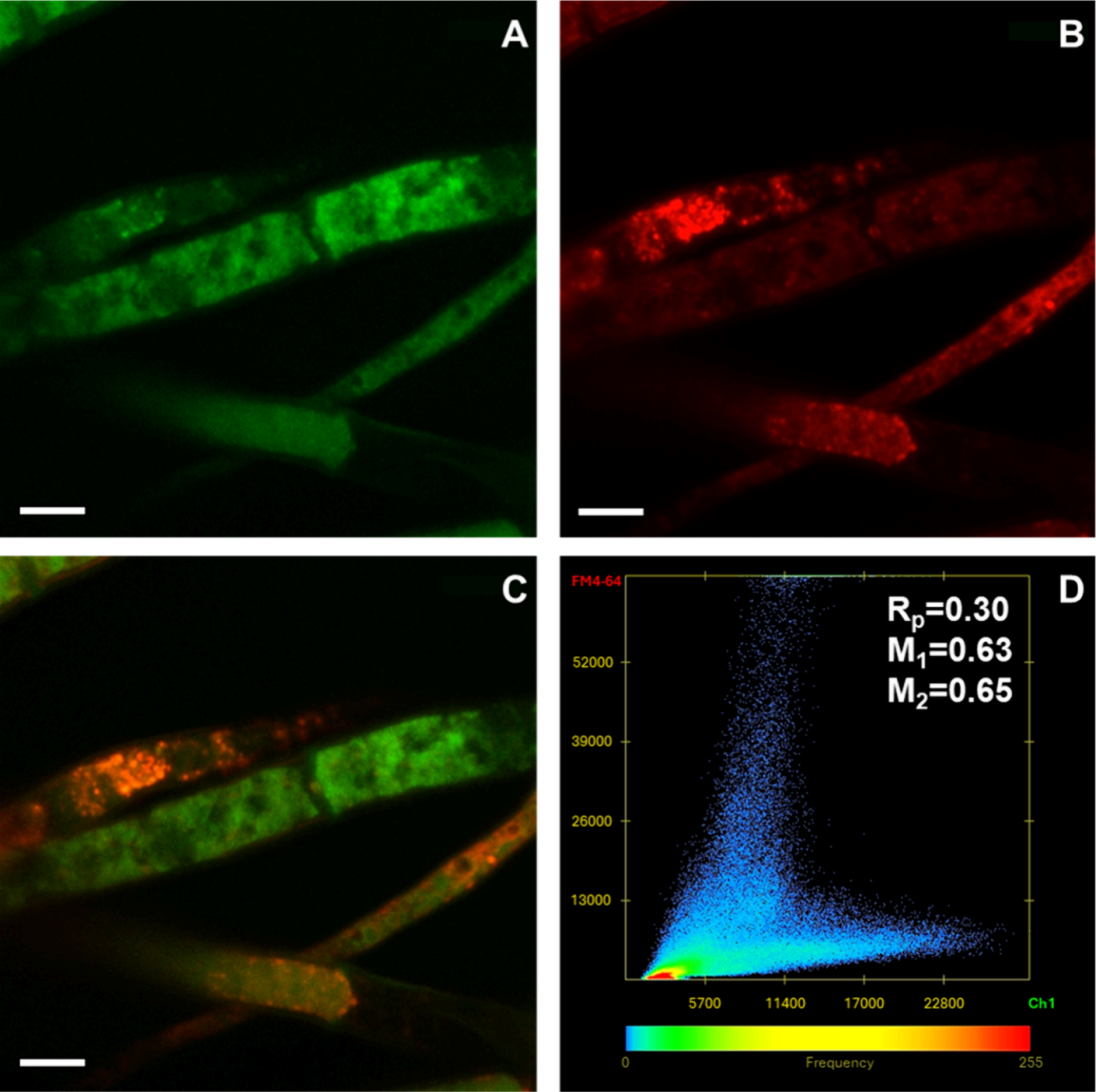
Colocalization analysis of CDs-NH_2_ with the endocytic probe FM4-64 in *B. cinerea* hyphae: (A) green fluorescence of CDs-NH_2_; (B) red fluorescence of FM4-64; (C) merged images. (D) Scatterplot of red versus green intensities generated using Zeiss Zen 3.1 software; Pearson (*R*_p_) and Manders (*M*_1_ and *M*_2_) coefficients are indicated. Scale bars: 5 μm.

Moving to plant cells, the penetration of CDs-NH_2_ in *Oryza sativa* was observed within a few minutes after incubation. At the beginning (≤5 min), the fluorescence signal was localized in the hairs of rice roots. The fluorescence was then transferred to the epidermal cells and concentrated in the thin cytoplasmic layer between the plasma membrane and the tonoplast (5–10 min after incubation) (Figure 5A). No fluorescence was observed within the vacuole. The CDs-NH_2_ signal was clearly observed in the nuclei of many epidermal cells (Figure 5B,C). By increasing the postincubation time (20 min), the signal was also observed in the vascular cylinder (Figure 5B). Microscopic analysis of the whole organ (not sectioned to avoid artifacts) did not allow to distinguish the tissues of the vascular cylinder involved in CDs-NH_2_ accumulation.

**Figure 5:**
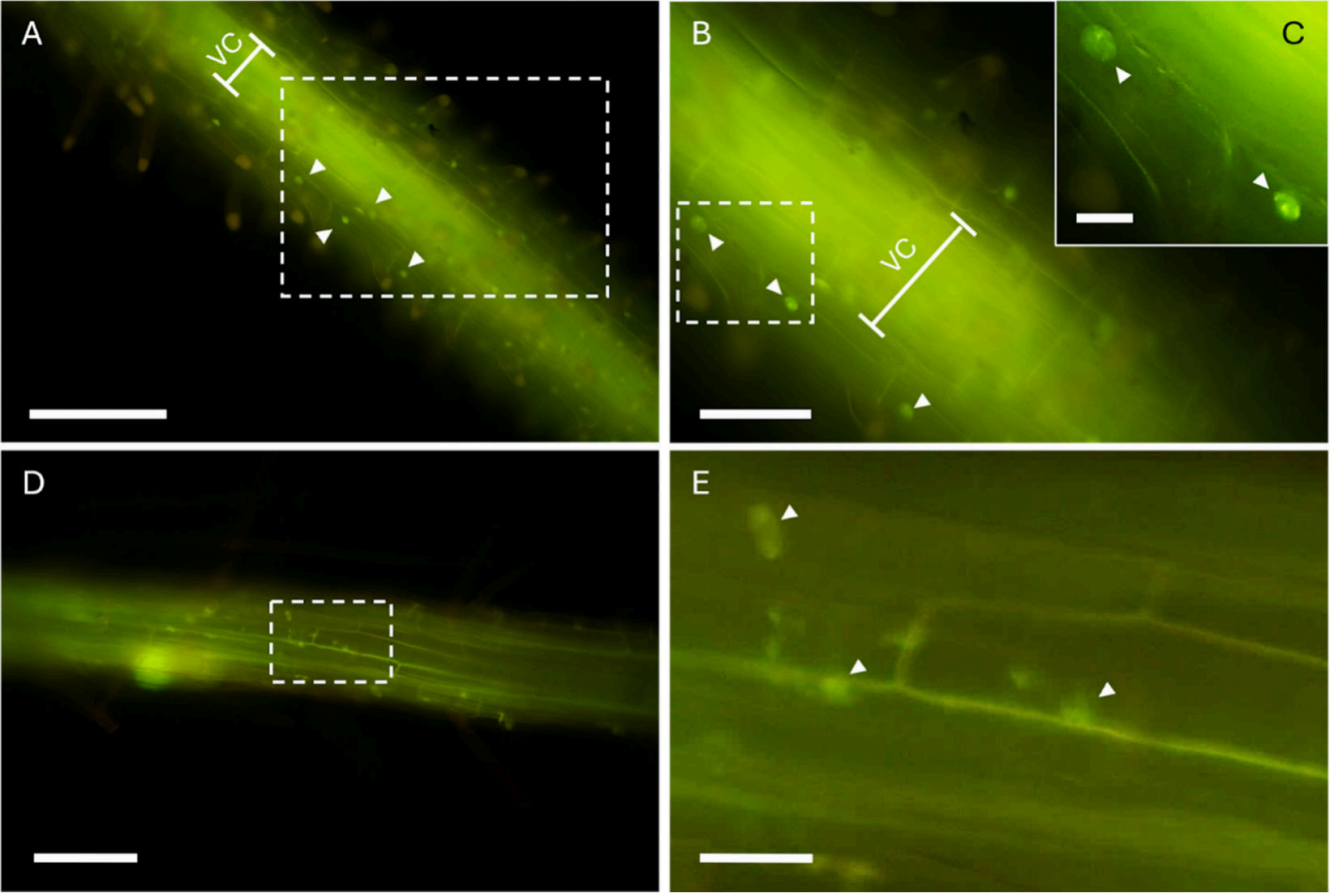
(A–C) Fluorescence images of whole rice (*O. sativa*) roots at the transition region between the elongation and maturation zones, treated with 0.5 mg/mL of CDs-NH_2_ and observed after 20 min and (D, E) 1 h. (A) A fluorescent signal is clearly visible in the vascular cylinder (VC) and in the nucleus of epidermal cells (arrowheads). To show the fluorescence of the nuclei in more detail, an enlargement of the area circumscribed by the dashed rectangle in (B) is shown in (C). (D) Fluorescence in the nuclei and in the cytoplasm of the epidermal cells. (E) Detail of the area bounded by the dashed rectangle in (D). Scale bars: (A, D) 500 μm; (B) 200 μm; (C) 50 μm; (E) 100 μm.

Fluorescence analysis is a standard strategy for investigating nanoparticle uptake, transport, and accumulation in biological systems. Unlike traditional nanoparticles that require external fluorophores such as coumarin 6,^45,46^ CDs are ideal for localization studies due to their intrinsic fluorescence. This property has enabled the use of confocal microscopy to track CDs in *Nicotiana tabacum* BY-2 cells,^47^ as well as to demonstrate their root-to-shoot translocation through the vascular system in mung bean plants,^48^ consistent with findings by Zhang et al.^49^

To complement these qualitative observations and gain a deeper understanding of CDs-NH_2_ uptake, quantitative fluorescence-based analyses were performed on rice roots, whose extended root system allowed for a clearer evaluation of nanoparticle internalization. Root adsorption has been recently investigated^50^ through the evaluation of fluorescence emission intensity by confocal microscopy. To obtain more reliable information, we decided to measure the decrease in the CDs-NH_2_ concentration over time in the external environment. The concentration of CDs-NH_2_ was assessed using a fluorescence calibration curve, based on emission intensity at 525 nm within a dynamic range of 5 to 25 μg/mL. The intrinsic fluorescence of CDs-NH_2_ enabled the detection of trace amounts in solution with high sensitivity. A strong linear correlation (*R* ^2^ = 0.9958) was observed between emission intensity and CDs-NH_2_ concentration, validating the method’s reliability (Figure S13). Application of this calibration curve revealed that the concentration of CDs-NH_2_ determined using eq 5 remained relatively stable during the initial hours of exposure (Table 1 and Figure S14). However, after 24 h, approximately 36% of the nanoparticles were no longer detectable in solution compared to the blank, suggesting significant adsorption. This reduction indicates a strong interaction with rice seedlings and likely internalization of the nanoparticles, as supported by fluorescence microscopy. These findings highlight the potential of CDs-NH_2_ for targeted delivery applications in plants and their possible use in addressing plant pathogen-induced diseases.

**Table 1:** Amount of CDs-NH_2_ Absorbed by Rice Roots at Different Time Points, As Determined by Fluorescence Measurements

time (h)	CDs-NH_2_ absorbed by rice seedlings (%)
0	3.7 ± 4.5
1	6.1 ± 4.7
2	2.1 ± 3.0
4	9.8 ± 4.5
24	36.2 ± 2.2

Given the possibility that CD-NH_2_ interacts with plant tissues, we investigated whether these nanomaterials could act as biostimulants or phytotoxins or remain biologically inert during the early stages of tomato growth. To this end, tomato seeds were incubated in CDs-NH_2_ solutions at various concentrations and then sown in a suitable culture medium. The germination rate was subsequently determined 3 and 7 days after sowing. As expected, the impact of the treatments was most pronounced on day 3, when a significant concentration-dependent promotion of germination was observed (Figure 6A). Treatment with 0.033 mg/mL CDs-NH_2_ led to a slight increase in the germination rate (37%) compared to the control (31%); however, this 6-percentage-point difference was not statistically significant. In contrast, the 0.066 mg/mL concentration significantly enhanced germination to 40% (a 9% increase over the control). The highest germination rate (64%) was achieved at the maximum concentration tested (0.132 mg/mL), representing more than a 2-fold increase relative to the control group. By day 7, differences between treatments were, as expected, less pronounced, as most seeds in all groups had successfully germinated. Nonetheless, it is noteworthy that germination rates remained significantly higher in seeds treated with 0.066 mg/mL (94%) and 0.132 mg/mL (98%) compared to the control group (88%), indicating a persistent, albeit attenuated, stimulatory effect.

**Figure 6:**
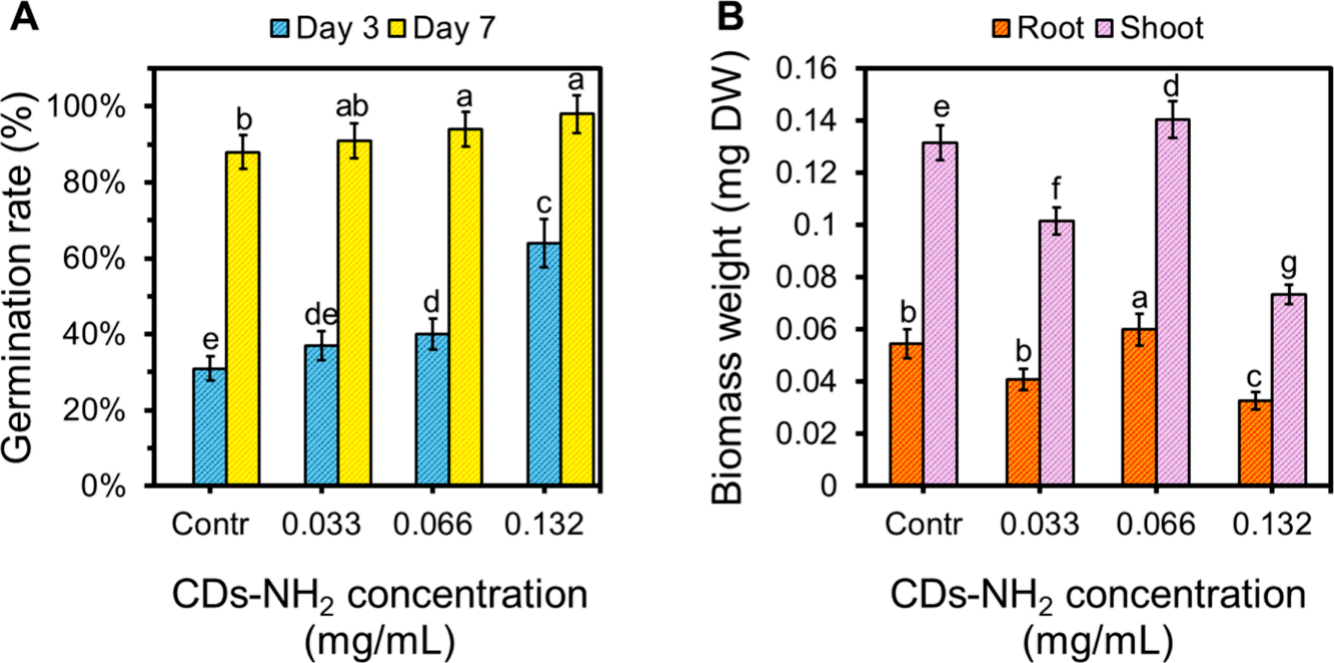
Dose–response effect of CDs-NH_2_ on tomato seed germination and seedling development. (A) Germination percentage and (B) root and shoot dry weight (DW) under various CDs-NH_2_ concentrations (0, 0.033, 0.066, and 0.132 mg/mL). Data are presented as mean ± SE of three independent replicates. Different letters indicate statistically significant differences between treatments (*P* < 0.05).

Seedlings derived from tomato seeds incubated with the aforementioned CDs-NH_2_ concentrations were harvested 11 days after sowing to evaluate the root and shoot dry biomass (Figure 6B). Significant increases in both root and shoot growth were observed in plants treated with 0.066 mg/mL CDs-NH_2_, which reached the highest biomass values (0.0599 and 0.1405 mg, respectively). Conversely, at the highest concentration tested (0.132 mg/mL), a significant reduction in biomass was detected compared to the control, with values dropping to 0.0328 mg for roots and 0.0734 mg for shoots. These results suggest that CDs-NH_2_ act as growth promoters at intermediate concentrations, consistent with previous findings for glucose- and l-cysteine-derived carbon dots in tomato and lettuce; however, higher doses may exert growth inhibition.^51^

Since seed germination is a crucial initial stage of plant development, ensuring its success is fundamental to optimizing overall plant growth.^52^ Recent studies have widely acknowledged the potential of CDs to enhance germination and early seedling development through various physiological mechanisms.^53^ Surface functionalization of CDs with hydrophilic groups (−OH, −COOH, and −NH_2_) significantly improves germination rates by boosting the imbibition capacity of seeds.^54,55^ These functional groups provide binding sites for water molecules, thereby facilitating their entry through the seed coat. Furthermore, CDs have been shown to upregulate the expression of aquaporin-related genes, which accelerate water transport and promote the elongation of roots and hypocotyls.^53–55^ Additionally, CDs exert a positive influence on fundamental plant processes, including root-mediated water and nutrient uptake, as well as photosynthetic efficiency.^51,53,55^

Recent research highlights that nitrogen doping of CDs significantly enhances their efficacy in promoting seed germination and plant growth. Beyond the aforementioned mechanisms, N-CDs can function as nanofertilizers by providing a slow-release source of bioavailable nitrogen. Chen et al. (2020)^56^ demonstrated across tomato, maize (*Zea mays*), and Pakchoi (*Brassica rapa* var. *chinensis*) that amino-functionalization confers superior performance regarding biomass accumulation, root cell elongation, chlorophyll content, and seedling transpiration. The advantageous effects of N-CDs have been reported across a broad range of species in a dose-dependent manner, including rice (*O. sativa*),^57^ soybean (*Glycine max*),^58^ pea (*Pisum sativum*),^59^ and mung bean (*Vigna radiata*).^60^

### 2.3 Analysis of the Stability of CDs-NH_2_ in Abiotic and Biological Environments

To evaluate the potential applicability of CDs-NH_2_, we investigated their short-term stability in abiotic and biotic media over 1 week, to assess whether exposure to these conditions alters the nanoparticle fluorescence and chemical composition.

Fluorescence was selected since it is the main tool to track the movement of the nanoparticles within biological microorganisms. The measurements were performed in both Milli-Q water and PBS, the latter being selected as an aqueous buffer solution of reference for biological assays. The emission spectra recorded upon excitation at 450 nm showed that the fluorescence remained nearly unchanged, as the overall intensity was of the same order of magnitude (Figure S15A,B). The slight variation in relative fluorescence intensity can be ascribed to evaporation of the aqueous media over the 7 days. FTIR spectra (Figure S15C) showed that the functional groups of the nanoparticles remained unchanged after treatment, indicating good chemical stability. We further recorded the ^1^H NMR spectrum, the technique being more sensitive to subtle variations in molecular connectivity and to the presence of small degradation products (Figure S15D). NMR spectra remained essentially unaltered, although a few isolated sharp peaks appeared, which may correspond to small molecular fragments released during the treatment. Finally, the TGA analysis confirmed that CDs-NH_2_ remained almost unaltered after 1 week, since the nanoparticles’ profiles displayed resembling trend (Figure S15E).

The intracellular stability of CDs-NH_2_ was evaluated via the fluorescence analysis of *Arabidopsis* root tissues following incubation. Observations revealed that fluorescence persisted for at least 7 days, suggesting that the CDs-NH_2_ (or at least a subpopulation of them) remained structurally intact throughout this period (Figure S16).

### 2.4 Antioxidant, Antifungal, and Antibacterial Activity of CDs-NH_2_

The *in vitro* antioxidant activity of CDs-NH_2_ was evaluated by using the DPPH and ABTS radical assays. These tests are widely used because they are nonspecific assays for assessing the overall antioxidant activity toward various electron-scavenging species. Although the predominant mechanism has not yet been unequivocally established, current evidence suggests that carbon dots can neutralize DPṖ and ABTṠ^+^ through both hydrogen-atom transfer (HAT) from surface functional groups and electron transfer (ET) from the carbonaceous core. These two pathways may even operate synergistically, with their relative contributions depending on the CDs’ surface chemistry and electronic structure. However, it is generally accepted that the HAT pathway is more limited, mainly due to steric hindrance and restricted accessibility of hydrogen-donating sites on the CDs surface (Figures S17 and S18).^61,62,15^

In light of this, CDs-NH_2_ were tested after one h of treatment in biologically relevant concentrations window from 15 to 250 μg/mL. CDs-NH_2_ demonstrated clear concentration-dependent radical scavenging activity in both DPPH and ABTS assays. Notably, the IC_50_ value decreased from 62.5 μg/mL at the initial time point to 31.2 μg/mL after 30 and 60 min, indicating enhanced activity over time (Figures S19 and S20).

The high antioxidant activity observed for CDs-NH_2_ can be rationalized by considering the widely accepted mechanism whereby enhanced radical-scavenging efficiency is associated with nanostructures rich in aromatic domains and hydrogen-containing functional groups. Such groups can readily donate hydrogen atoms (characterized by lower bond dissociation energies (BDEs) compared to C–H bonds) and stabilize the resulting radical through delocalization within the aromatic framework.^15^ The high antioxidant activity displayed by CDs-NH_2_ may suggest their potential as protective nanoagents for plant crops, particularly in preventing or reducing oxidative stress generated during infections by phytopathogens.

The *in vitro* and *in planta* antimicrobial activity of CDs-NH_2_ was assessed against representative fungal and bacterial pathogens of tomato plants, namely, *B. cinerea* and *P. syringae* pv. *tomato*. The antifungal activity of CDs-NH_2_ was first assessed, revealing a MIC_50_ value higher than 0.500 mg/mL, indicating no significant inhibition of *B. cinerea* planktonic growth (data not shown). Subsequently, the ability of CDs-NH_2_ to inhibit biofilm formation was assessed using five concentrations ranging from 0.0312 to 0.500 mg/mL. As shown in Figure 7A, a dose-dependent reduction in biofilm formation was observed, with approximately 50% inhibition achieved at a concentration of 0.125 mg/mL.

**Figure 7:**
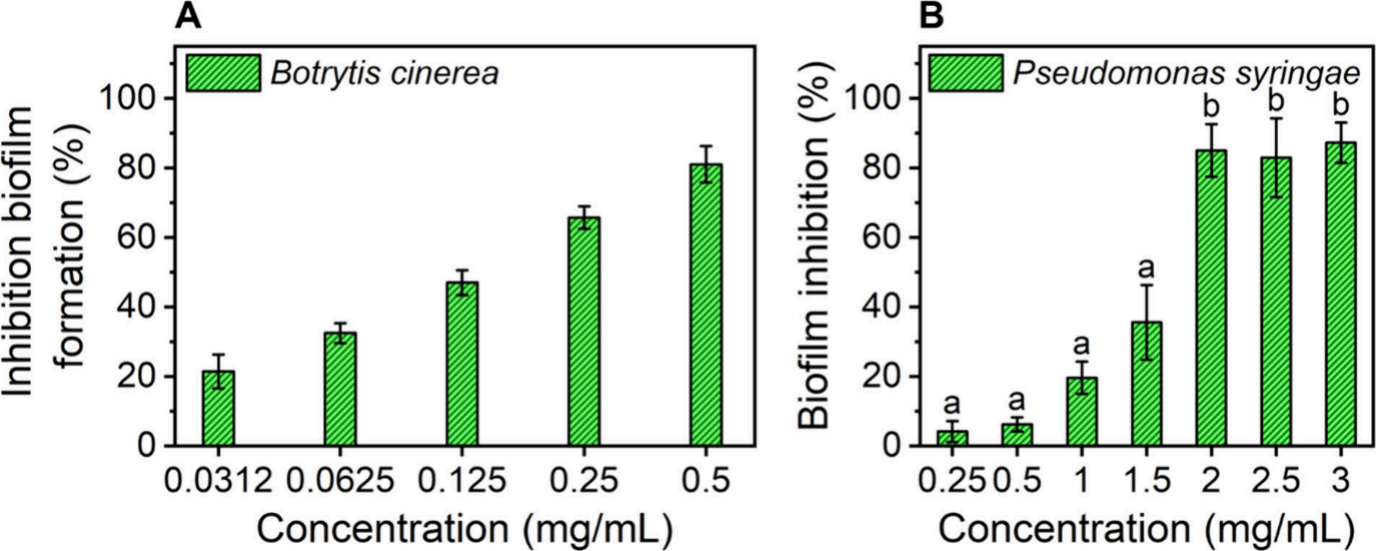
Antibiofilm activity of CDs-NH_2_ on (A) *B. cinerea* and (B) *P. syringae*. (A) Inhibitory activity of CDs-NH_2_ on *B. cinerea* biofilm formation. Each data point represents the mean of three independent experiments ± SE. All group comparisons showed statistically significant differences with *p*-values of <0.0001 (Tukey’s test). (B) Effect of different concentrations of CDs-NH_2_ on *P. syringae* biofilm inhibition after 24 h of incubation, normalized to the control (PtoDC3000 suspension in LB medium alone); error bars indicate SE. Different letters (a, b) show significantly different values after one-way ANOVA, followed by Tukey’s HSD post hoc test (*p* < 0.05).

The data presented demonstrate that CDs-NH_2_ do not exert growth inhibitory activity; instead, they act by inhibiting a key virulence factor at nontoxic concentrations.^29^ Antivirulence strategies are increasingly recognized as promising approaches to reduce disease severity while minimizing selective pressure for resistance and preserving the beneficial microbiota.^63^

Given the promising results in inhibiting fungal biofilms, we further explored the antibacterial properties of CDs-NH_2_ to assess their overall efficacy against *P. syringae* pv. *tomato*, a bacterial pathogen affecting tomato plants. The antibacterial activity was first evaluated through the agar diffusion assay, by measuring the diameter of the inhibition zones at concentrations ranging from 0.25 mg/mL to 10 mg/mL. The minimum inhibitory concentration (MIC) of CDs-NH_2_ was found to be 5 mg/mL. No bacterial inhibition zone was noticed at lower concentrations and larger diameter of inhibition zone was observed at higher concentration of CDs-NH_2_ (Figure S21A,B). Accordingly, the antibacterial activity of CDs-NH_2_ was found to be highly concentration-dependent. Subsequently, the ability of CDs-NH_2_ to inhibit biofilm formation was evaluated. Interestingly, it was found that CDs-NH_2_ significantly inhibited biofilm formation at a concentration of 2.0 mg/mL, lower than that required to inhibit bacterial growth (Figure 7B).

Microbiostatic activity against both bacterial and fungal pathogens was evident only at higher concentrations of CDs-NH_2_, whereas substantial inhibition of biofilm formation was achieved at significantly lower concentrations. Interestingly, similar results were observed in *C. albicans* treated with the same type of CDs.^34^ Indeed, CDs-NH_2_ efficiently internalized into *C. albicans* cells, inhibited adhesion and biofilm formation, and displayed *in vivo* efficacy with negligible toxicity, supporting a close correlation between surface charge, cellular uptake and antifungal activity.^34^ In line with these findings, in the present study, CDs-NH_2_ showed rapid accumulation on the cell wall of *B. cinerea*, followed by internalization into vesicle-like intracellular structures and a marked inhibition of biofilm formation at concentrations that do not significantly affect planktonic growth. This pattern suggests an antivirulence-type mechanism, whereby CDs-NH_2_ primarily interferes with adhesion and biofilm development rather than acting as classical fungicidal agents. In this way, pathogenicity is alleviated without microbial eradication, thus contributing to the preservation of the host-associated microbiota’s structure and functionality.

Biofilm formation represents a fundamental virulence trait of many pathogenic fungi. Most mycoses are strongly correlated with biofilm development on biotic or abiotic substrates. Biofilm maturation facilitates hematogenous dissemination and constitutes a critical determinant of the high morbidity and mortality rates observed in invasive candidiasis.^64^

Overall, these findings align with recent studies which have reported that positively charged carbon dots can exert antifungal activity through multiple mechanisms, including electrostatic interactions with the fungal cell wall, perturbation of the cell envelope and, in some cases, oxidative stress-related effects.^28^

To further investigate antibacterial efficiency *in planta*, CDs-NH_2_ were applied to tomato transplants, and divided into four groups, i.e., nontreated plants (TL H_2_O), CDs-NH_2_-treated plants (TL CDs-NH_2_), bacterial-infected plants (TL Pto) and bacterial-infected CDs-NH_2_-treated plants (TL CDs-NH_2_+Pto). Subsequently, leaves from grown plants were in part observed to assess the severity of symptoms and in part collected for the metabolomic analysis (see section 2.5).

The severity of the disease, ranked between 0 for leaves with no symptoms, 1 in case of few water-soaked lesions with halo, 2 pinpoint lesions (<1 mm) and 3 for evident necrotic dots (>1 mm), was recorded 7 days after inoculation (Figure 8). All the tomato plants treated with H_2_O and CDs-NH_2_ as negative control showed no symptoms as expected and were scored as (0). Among the 20 plants inoculated with *P. syringae*, 12 and 8 were scored, respectively, as (2) and (3), corresponding to an overall disease severity of 80%. When the infected plants were treated with CDs-NH_2_, the bacterial speck severity significantly lowered from 80% to 41%. In fact, 2, 11, and 7 plants were scored, respectively, as (0), (1) and (2), indicating that CDs-NH_2_ were effective in inhibiting *P. syringae* infection *in planta*.

**Figure 8:**
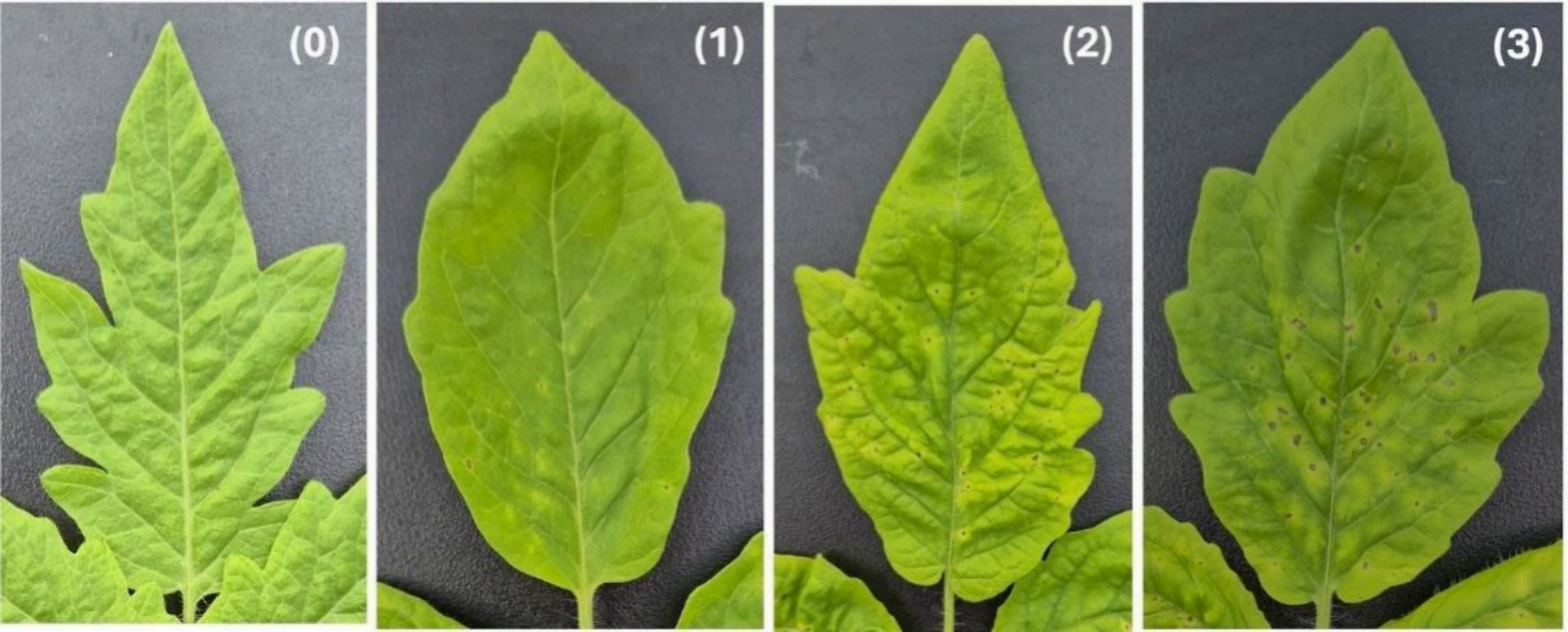
Representative leaves belonging to disease severity scale: (0) no symptoms; (1) few water-soaked lesions with halo; (2) pinpoint lesions <1 mm surrounded by yellow halo; (3) evident necrotic dots >1 mm, chlorosis and coalescence area.

These *in planta* results agree with previous *in vitro* studies: indeed, the antimicrobial activity of CDs is well-known and widely studied during recent years against many microorganisms. Specifically, CDs synthesized from citric acid and β-alanine have shown inhibition of *P. syringae* growth, with the minimum inhibitory concentration (MIC) of 5 mg/mL,^65^ data which are in accordance with our results. Devkota et al.^66^ reported that *Pseudomonas* growth was inhibited at 0.5 mg/mL, whereas for *Agrobacterium*, *Salmonella*, *Pectobacterium*, and *E. coli* at least 5 mg/mL was needed to observe growth inhibition *in vitro*. Earlier reports confirm that the inhibitory effects of CDs on bacterial growth highly depend on composition, size, shape, surface charge, and chemistry of the CDs, as well as the structure and surface chemistry of microorganisms.^67^ Less is known about the reduction of disease severity caused by *P. syringae* pv. *tomato* on tomato plants prior to treatment with CDs. The *in vivo* test performed in this study provided encouraging results on the contribution of CDs in reducing disease severity when applied to plants prior to bacterial infection. Similar results have been obtained by Luo et al.:^68^ foliar application of N-CDs has been shown to significantly reduce disease severity by suppressing bacterial wilt in tomatoes caused by *Ralstonia solanacearum*.

### 2.5 ^1^H NMR-Based Metabolomics Analysis of Tomato Leaves in Response to *P. syringae* pv. *tomato* Infection and CDs-NH_2_ Treatment

To evaluate the effect of *P. syringae* pv. *tomato* infection both in the presence and absence of CD-NH_2_, tomato leaf samples were analyzed by an ^1^H NMR based metabolomic approach. Briefly, each sample was collected and extracted following a modified Bligh and Dyer method.^69^ From the collected hydroalcoholic and lipophilic extracts, in total, 48 metabolites were identified and quantified. Chemical shift, resonance assignment and signal multiplicity are reported in Table S2. The quantified molecules, listed in Table S3, were found belonging to several categories, including amino acids, carbohydrates and polyols, organic acids, lipids and sterols, and other compounds. Reported in Table 2 are metabolites whose significance was observed with the ANOVA test.

**Table 2:** Quantification of Metabolites Showing Statistically Significant Differences Compared to the Controls (TL H_2_O), Based on ^1^H NMR Spectra and Evaluated by the ANOVA Test^a^

	amount (mg/100 g)			
metabolite	TL H_2_O	TL CDs-NH_2_	TL Pto	TL CDs-NH_2_+Pto
Amino Acids				
Aspartic acid	5.46 ± 0.64	7.35 ± 2.72	17.23 ± 3.33**	13.07 ± 1.83*
Isoleucine	0.24 ± 0.06	0.25 ± 0.05	1.35 ± 0.55*	1.01 ± 0.35
Glutamic acid	20.29 ± 0.97	20.31 ± 2.79	28.23 ± 4.7*	26.62 ± 1.53
Glutamine	13.58 ± 0.78	13.85 ± 1.64	24.38 ± 5.88*	20.36 ± 2.84
Glycine	7.86 ± 1.57	9.95 ± 0.82	17.35 ± 3.65*	14.42 ± 2.61
Leucine	0.24 ± 0.03	0.16 ± 0.02	1.82 ± 0.79*	1.15 ± 0.48
Valine	0.44 ± 0.06	0.49 ± 0.01	1.86 ± 0.77**	1.07 ± 0.33
Carbohydrates and Polyols				
Glucose	9.07 ± 1.9	10.57 ± 2.53	5.27 ± 1.87	4.42 ± 0.24*
Sucrose	39.85 ± 11.12	54.81 ± 4.38	94.13 ± 20.02*	76.25 ± 13.57
U01 (polygalacturonic acid)	5.47 ± 1.61	4.73 ± 1.18	2.24 ± 1.28*	1.36 ± 1.14**
Organic Acids				
Chlorogenic acid	2.5 ± 0.3	2.3 ± 0.67	1.04 ± 0.7*	0.41 ± 0.53**
Citric acid	166.06 ± 11.56	152.23 ± 37.63	124.33 ± 20.16	79.44 ± 47.29**
Malic acid	49.4 ± 6.96	51.19 ± 9.40	36.06 ± 3.95	20.86 ± 13.05*
Neochlorogenic acid	5.48 ± 1.46	5.43 ± 1.48	2.63 ± 1.24	1.3 ± 1.15*
U02 (Caffeoyl malic acid)	12.02 ± 2.85	11.03 ± 3.71	4.54 ± 2.7*	1.97 ± 2.71**
Lipids and Sterols				
Omega 3 FA	61.31 ± 11.51	53.02 ± 6.06	46.87 ± 9.49	33.16 ± 10.51*
Total unsaturated fatty acids	84.23 ± 12.17	70.41 ± 5.46	66.98 ± 11.4	52.57 ± 17.37*
Other Compounds				
Allantoin	5.47 ± 0.94	6.76 ± 1.28	7.92 ± 1.26	8.73 ± 1.28*

a The significant variables of the ANOVA test were indicated with * for the significant differences in one way-ANOVA against controls (TL H_2_O) with *p* < 0.05, and with ** for the significant differences in one way-ANOVA against controls (TL H_2_O) with *p* < 0.01. Multiple groups comparison was corrected with the Dunnett test.

Tomato leaves treated with CDs-NH_2_ showed no significant metabolic differences compared to TL H_2_O treatment. The results obtained from the univariate analysis showed mainly differences between TL Pto and TL CDs-NH_2_+Pto treated samples against the control group (TL H_2_O). An increase in amino acid levels (including aspartic acid, isoleucine, glutamic acid, glycine, glutamine, leucine, and valine) was observed in TL Pto. Among these, aspartic acid levels were significantly higher in TL CDs-NH_2_+Pto than in the control samples. As per carbohydrates and polyols, higher levels of sucrose were observed in TL Pto, while lower quantities of glucose were measured in TL CDs-NH_2_+Pto compared with control samples. U01 (polygalacturonic acid) levels decreased in pathogen-treated samples independently of the presence of CDs-NH_2_, and a similar trend was observed for organic acids such as chlorogenic acid and U02 (caffeoyl malic acid). Citric acid, malic acid, and neochlorogenic acid levels decreased only in the Pto+CDs-NH_2_ group, compared to control. Uniformly, a reduction in omega-3 and total unsaturated fatty acids was observed in the CDs-NH_2_+Pto group, while allantoin content increased compared to control samples. To explore the relationships among the multiple variables, a PCA analysis (principal component analysis) was performed (Figure 9A,B) and significant variables were discussed.

**Figure 9:**
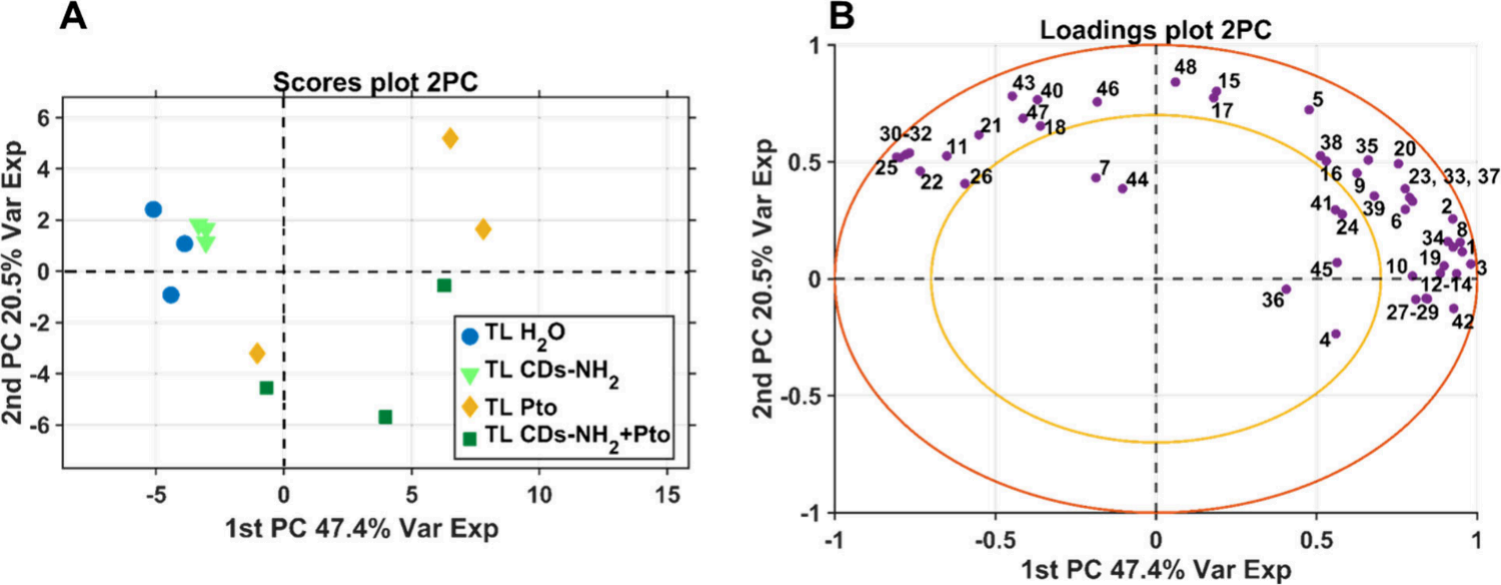
(A) PCA scores plot for the first two principal components of tomato leaf samples, H_2_O, CDs-NH_2_, Pto, and CDs-NH_2_+Pto. (B) PCA loadings plot for the first two principal components of tomato leaf samples, H_2_O, CDs-NH_2_, Pto, and CD-NH_2_+Pto. The red ellipse represents the 100% variance ellipsoid, while the yellow one represents the 50% variance ellipsoid. 1. Leucine, 2. valine, 3. isoleucine, 4. 2,3-butanediol, 5. threonine, 6. alanine, 7. quinic acid, 8. glutamine, 9. GABA, 10. glutamic acid, 11. citric acid, 12. asparagine, 14. malonic acid, 15. ethanolamine, 16. choline, 17. betaine. 18. myo-inositol, 19. glycine, 20. chiro-inositol, 21. total fructose, 22. malic acid, 23. dihydroxyacetone, 24. ascorbic acid, 25. U01 (polygalacturonic acid), 26. glucose, 27. allantoin, 28. sucrose, 29. uridine moiety, 30. chlorogenic acid, 31. neochlorogenic acid, 32. U02 (caffeoyl malic acid), 33. tyrosine, 34. tryptophan, 35. 4-hydroxybenzoic acid, 36. formic acid, 37. trigonelline, 38. β-sitosterol, 39. camposterol, 40. total unsaturated fatty acids, 41. total saturated fatty acids, 42. omega 6 fatty acids, 43. omega 3 fatty acids, 44. omega 9 fatty acids, 45. glycerophospholipids, 46. total carotenoids, 47. phaeophytin a and 48. phaeophytin b.

In PCA the experimental factor between treatments is observable along the first PC (principal component); 67.7% of variance of the data matrix is explained on the first 2 PC (Figure 9A,B and Figure S22). No PCA outliers are found from the influence plot, considering the T squared and Q residual values (Figure S23). TL H_2_O and TL CDs-NH_2_ samples all reside in the negative side of the axis, while TL Pto and TL CDs-NH_2_+Pto reside all on the positive part (except for one sample for both Pto and CDs-NH_2_+Pto treatment that are close to the 0 axis, as reported in Figure 9A). Those differences are mainly accounted by all caffeic acid esters (chlorogenic, neochlorogenic acids and U02), fructose, glucose, myo-inositol, carotenoids phaeophytin a, and total unsaturated fatty acid and omega 3 fatty acid loading coefficients (in order of negative contribution to the first PC), of which are above-mean value for TL H_2_O and TL CDs-NH_2_ samples. Similarly, for TL Pto and TL CDs-NH_2_+Pto above-mean value of loadings coefficients are reported for valine, isoleucine, leucine, glycine, glutamine, glutamic acid, asparagine, aspartic acids, malonic acid, allantoin, uridine moiety, alanine, tyrosine and GABA (in order of positive contribution to the first PC) (Figure 9B).

The untargeted metabolomics approach employed in this study allowed us to observe the metabolic impact of CDs-NH_2_ treatment in tomato leaves and their effect in the presence of common pathogenic affliction with *P. syringae* pv. *tomato*. Interestingly, the treatment with CDs-NH_2_ clusters tomato leaves closely with control samples, as shown in the score plot in Figure 9A, indicating no CDs-NH_2_-induced metabolic alteration. In other words, noninfected leaves treated with CDs-NH_2_ present a nonsignificant variation of quantities in all metabolites compared to controls (TL H_2_O), evidence that there are no basal metabolic shifts and possible nontoxicity of the treatment (Table 2). As per unsupervised multivariate analysis, the main impact on tomato leaf metabolism stems from the presence of the pathogen. As revealed by the trend of free amino acids, an increase in aliphatic compounds was induced by treating the leaves with *P. syringae* pv. *tomato*. As previously observed in tomato plants, the increase of free amino acids could be linked to a protein turnover happening in infected leaves, dealing with the damage of the infection.^70^ Interestingly, a 9-fold increase is reported for free leucine in CDs-NH_2_-untreated infected leaves, which may be linked to the turnover of leucine-rich proteins that are known to be involved in infections and defense.^70^ Upon infection, a metabolic reprogramming in the primary metabolism, involving amino acids directly derived from the TCA (tricarboxylic acids) cycle is known.^71^

A remarkable effect of CDs-NH_2_ on TCA cycle of infected leaves (CDs-NH_2_ Pto) is observable by the lower quantities of citric acid and malic acid compared to Pto group, expressing different metabolic fluxes toward energy usage and synthesis. Specific metabolic alterations of aspartic acid and glutamic acid derivatives during *P. syringae* pv. *tomato* infections were already described in literature.^71^ Glutamic acid and aspartic acid are the two main amino acids that can be further used to synthesize other amino acids. In our study, in infected tomato leaves, aspartic acid levels dramatically increased, which was noticeably lowered by treatment with CDs-NH_2_ suggesting an alleviating effect of CDs-NH_2_. However, since the role of glutamic and aspartic acids and their amides is also found to be nitrogen storage and assimilation, a stress response could be inferred between the pathogen and the hosting plants with the presence of the CDs-NH_2_.^72^ A shift in nitrogen usage and a different stress modulation undergoing in CDs-NH_2_ infected tomato leaves is suggested by the higher quantities of allantoin, a well-known compound belonging to purine metabolism, as reported in Table 2.

In fact, the combined effect of pathogen and CDs-NH_2_ seems to influence the metabolic pathway of the purine metabolism, differently from the solely pathogenic infection that affected the aspartate and glutamate pathway.^73^ The role of allantoin accumulation in the state of a pathogenic attack is linked to the activation and signaling of the jasmonates pathway, acting as a primary defense mechanism.^74^

A differential carbohydrate response was observed in infected leaves, as revealed from the decrease of free carbohydrates glucose and fructose, while sucrose content greatly increased compared to control samples. This may be imposed to share energy usage and competition between the plant and the bacterial infection. As shown in Table 2, this trend was observed also in infected tomato leaves treated with CDs-NH_2_, as per glucose, significantly lower in CDs-NH_2_ samples, and sucrose, instead significantly lower in infected only leaves. It is important to note that given the enzymatic contribution of *P. syringae* pv. *tomato* on tomato leaves, competitive usage on carbon resources cannot be excluded.^75^ Since no significant sucrose level differences were found in infected tomato leaves after treatment with CDs-NH_2_ compared to control samples, a contribution of CDs-NH_2_ on sugar modulation can be suggested.

Moreover, our results evidenced the role of CDs-NH_2_ on lipid metabolism of tomato leaves in the presence of the pathogen (CDs-NH_2_+Pto) as revealed by the significant decrease in total unsaturated fatty acids and omega 3 levels, compared to control samples (see Table 2). This trend, paired with the observed higher variance in saturated fatty acids in all infected leaves (Figure 9B), could be due to a modulation in cell membrane fluidity and plasticity,^76^ further influenced by the presence of CDs-NH_2_. In plants, lipids are involved in structural and storage roles, as well as in the response mechanisms to biotic and abiotic stress.^76^

The impact of the infection in infected tomato leaves is also evidenced by a drop in all chlorogenic acid derivatives; those levels were even lower than observed in CDs-NH_2_-treated-infected leaves. It is well-known that phenols play a crucial role in plant defense mechanisms, exerting inhibitory effects on the growth of several bacterial and fungal pathogens, by inducing membrane and cell wall permeabilization and cell death.^77^

Overall, the treatment of tomato leaves with CDs-NH_2_ highlighted a reactive metabolic shift happening exclusively after infection. The observed metabolic differences involved in tissue plasticity and mobility, biosynthesis of amino acids, sugar metabolism, differential nitrogen storage, and accumulation of allantoin could pose a stress modulation and challenge to the pathogenic organism attacking the plant. It is thus evidenced that the CDs-NH_2_ priming in infected leaves occur mainly in primary metabolism, reestablishing, during infection, metabolic fluxes toward previous control and ‘healthy’ conditions. While those changes in amino acid, nitrogen storage, and sugar metabolism are happening, a defense response in the presence of the pathogen is also evidenced by alterations in the chlorogenic acid metabolism. A more direct defense and antimicrobic action of infected leaves may also be linked to the physical presence and properties of CDs-NH_2_. This joint collaboration between metabolic priming and antimicrobial activity may help plants to better adapt to possible challenges during infection, especially in vital resource usage.

Nonetheless, we applied for the first time a ^1^H NMR based metabolomics approach to observe the effect of a common pathogenic affliction in combination with CDs-NH_2_ treatment.

## 3 Conclusions

Amino-functionalized carbon dots (CDs-NH_2_) emerged in this study as promising nanomaterials for sustainable crop protection. A thorough investigation on the CDs-NH_2_ structural and morphological features was achieved by combination of NMR-DOSY, AFM, TEM, EDS, XRD, and Raman spectroscopy, setting the foundations for subsequent correlation between physicochemical properties and biological activities. After characterization, CDs-NH_2_ confirmed their high purity, intrinsic fluorescence, stability, and amino-rich surface.

CDs-NH_2_ demonstrated the ability to penetrate both plant and fungal cells and promoted seed germination and early seedling growth. In the presence of pathogens, CDs-NH_2_ displayed clear antimicrobial activity by mainly inhibiting the biofilm formation of *P. syringae* pv. *tomato* and *B. cinerea*, with minor effects on planktonic growth. *In planta*, treatment with CDs-NH_2_ notably reduced the disease severity caused by bacterial infection.

^1^H-NMR-based metabolomics analysis revealed that CDs-NH_2_ does not cause metabolic alterations in healthy tomato leaves, indicating their biocompatibility and lack of phytotoxicity. However, in pathogen-infected plants, CDs-NH_2_ induced specific metabolic reprogramming, partially reestablishing control conditions. This involved key pathways related to amino acid biosynthesis, sugar and lipid metabolism, nitrogen storage, and allantoin accumulation, suggesting an active role in modulating plant stress responses under biotic pressure.

This study presents the first NMR-based metabolomics investigation of the interaction between CDs and plant-pathogen systems. The dual action of CDs-NH_2_, as both antimicrobial agents and metabolic modulators in the presence of the pathogen, highlights their potential as innovative and environmentally friendly nanotools in integrated pest management.

Further investigations are currently underway to expand the omics-based analyses to fungal infections, including *B. cinerea*, and to examine the pathogen metabolic response to CDs-NH_2_ exposure. A deeper understanding of the mechanisms of action, particularly through integrated metabolomics, proteomics, and transcriptomics, will be essential to optimizing nanoparticle design for improved specificity and biological performance. Establishing structure–activity relationships and clarifying the molecular interactions between CDs-NH_2_ and pathogenic microorganisms will be essential for advancing their application in precision agriculture.

Regarding the safety profile of CDs-NH_2_, the evidence derived from this study and prior investigations^29,34^ indicates that, at the tested concentrations, these nanoparticles exhibit negligible toxicity. Our findings align with previous reports on amino-functionalized CDs.^78^ However, potential widespread agricultural applications necessitate exhaustive toxicological assessments across a broader range of bioindicators. Environmental fate and long-term biosafety of CDs-NH_2_ still remain key aspects for their employment.^79–81^ Overall, this work provides mechanistic insight and preliminary guidelines for designing biocompatible nanoplatforms to support integrated and sustainable disease-management strategies.

## 4 Materials and Methods

### 4.1 Synthesis and Characterization of CDs-NH_2_

#### 4.1.1 Materials

All reagents and solvents, except for HPLC-grade water (acquired from Honeywell), were purchased from Sigma-Aldrich and used without further purification. Syringe filters (100 nm pore size) were also obtained from Sigma-Aldrich. Dialysis membranes with a molecular weight cutoff (MWCO) of 100–500 Da were purchased from Carlo Erba.

#### 4.1.2 Synthesis of CDs-NH_2_

CDs-NH_2_ were synthesized following a previously reported protocol,^34^ with minor modifications in the purification steps. Briefly, d-(+)-glucosamine hydrochloride **1** (1.00 g, 4.64 mmol) was dissolved in 20 mL of Milli-Q water in a 250 mL conical flask, and the mixture was stirred to obtain a homogeneous solution. A separately prepared solution of 1,3-diaminobenzene **2** (0.55 g, 5.09 mmol) in 10 mL of methanol was sonicated for 10 min at 25 °C and then added to the glucosamine solution under continuous stirring. The reaction mixture was stirred for a few minutes before being subjected to microwave irradiation at 800 W for 3 min in a domestic microwave oven. Following the reaction, the resulting dark brown slurry was diluted with 20 mL of Milli-Q water, transferred into a falcon tube, and centrifuged at 4000 rpm for 45 min. The supernatant was then filtered through 100 nm syringe filters and subjected to dialysis using membranes with a molecular weight cutoff (MWCO) of 100–500 Da. The dialysis process was carried out in a 150 mM NaCl solution for 2 days, followed by three additional days in pure distilled water to remove salts. The dialysis water was replaced at least five times per day to ensure thorough purification. After dialysis, the solution was concentrated under reduced pressure using a rotary evaporator. Then, it was sonicated, frozen in liquid nitrogen, and lyophilized and 250 mg of a light brown, fluffy powder was obtained, corresponding to a mass yield of 16%.

#### 4.1.3 Kaiser Test

The Kaiser test was performed according to a modified literature protocol using a commercially available kit provided by Merck.^38^ Approximately 1 mg of CDs-NH_2_ was dissolved in 1 mL of Milli-Q water, and 250 μL of this solution was transferred to each of three separate vials. To each vial, 75 μL of a phenol solution in ethanol and 100 μL of a potassium cyanide (KCN) solution in pyridine/water were added. The mixtures were sonicated for 5 min at room temperature, after which 75 μL of a ninhydrin solution in ethanol were added. A blank solution was prepared following the same procedure, in which 250 μL of Milli-Q water were used in place of the sample solution. The sample and blank solutions were then heated at 120 °C for 5 min, then allowed to cool to room temperature for an additional 5 min. After the reaction, all four vials (three samples and one blank) were diluted with 2 mL of a diluting solution (60% ethanol:40% water). Then, 250 μL of each resulting solution were transferred into polystyrene cuvettes (1 cm path length) and further diluted with 2.5 mL of the same diluting solution. The absorbance spectra were acquired using a UV–vis V730 Jasco spectrophotometer over the 800–400 nm range, with a scan speed of 200 nm/min, and the blank solution was used as reference. The number of primary amino groups was estimated using eq 1:

1 $$KT\left(\frac{{\mathrm{\mu mol}}_{{\mathrm{NH}}_{2}}}{g_{{\mathrm{CDs‐NH}}_{2}}}\right)=\frac{A\cdot 10^{6}}{c\cdot \varepsilon \cdot l}$$

Equation 1 is used for the calculation of primary amino groups in CDs-NH_2_, where *A* is the absorbance of the cuvette solution recorded at 565 nm, *c* is the concentration of the stock solution (g/L), ε is the molar extinction coefficient (15 000 M^–1^·cm^–1^ for Ruhemann’s purple), and *l* is the path length of the cuvette (cm).

#### 4.1.4 Quantification of Surface Primary Amines on CDs-NH_2_ by ^19^F NMR

Primary amino groups of CDs-NH_2_ were additionally quantified through derivatization with 4-fluorobenzaldehyde (FBA). The reaction was carried out by dissolving CDs-NH_2_ (6.0 mg) in DMSO-*d*_6_ (0.6 mL), yielding a final concentration of 10 mg/mL. Then, FBA (7.45 mg, 60 μmol) was added dropwise, and the resulting mixture was stirred at room temperature for 24 h, protected from light. A parallel blank reaction was carried out under identical conditions in the absence of CDs-NH_2_. The ^19^F NMR spectrum of the reaction mixture was then acquired, and the number of primary amine groups was calculated by comparing the integral of the broad signals centered at –108.5 and –114.9 ppm to the residual signal of the aldehyde according to eq 2.

2 $$\frac{{\mathrm{mmol}}_{{\mathrm{NH}}_{2}}}{g_{{\mathrm{CDs‐NH}}_{2}}}=\frac{{\mathrm{\mu mol}}_{\mathrm{FBA}}}{{\mathrm{mg}}_{{\mathrm{CDs‐NH}}_{2}}}\cdot \frac{I_{\mathrm{Imine}}}{\overset{n}{\underset{i=1}{\sum }}I_{i}}$$

Equation 2 is used for the quantification of primary amino groups on CDs-NH_2_ determined by ^19^F NMR analysis, expressed as mmol of primary amino groups per gram of CDs-NH_2_. This value was obtained by dividing the number of μmoles of FBA by the mass (mg) of CDs-NH_2_ in the reaction mixture and multiplying by the integral of the imine signals, normalized to the total protons integral.

#### 4.1.5 NMR-DOSY Measurements

The NMR-DOSY experiments over CDs-NH_2_ were performed on a solution of 10 mg/mL in D_2_O using a Bruker Avance NEO 400 Nanobay NMR (operating at a frequency of 400 MHz). DOSY experimental setup values were initially optimized, setting a Δ value of 59.9 ms and a δ value of 3.64 ms. The acquisition parameters were set as *d*_1_ = 10 s, 32 points in the diffusion dimension, NS = 128 for each diffusion step, and *T* = 308.0 K (controlled by an external temperature controller unit). DOSY spectra were processed from the raw data using MestReNova v12.0.0-20080.

Diffusion coefficients were determined by fitting the exponential decay of the intensity of the corresponding peaks, using the diffusion fit function (eq 3). Additional details on diffusion coefficients, fit parameters, fit function display, and DOSY spectra are provided in Supporting Information (Figure S6A,B).

3 $$I\left(g\right)=I_{0}e^{-\left[g^{2}\delta^{2}\gamma^{2}\left(\Delta -\frac{\delta }{3}\right)D\right]}$$

Equation 3 is used to fit the diffusion decay in DOSY NMR experiments, where *I* is the signal intensity, *I*_0_ is the initial intensity, *g* is the gradient strength, δ is the gradient duration, γ is the gyromagnetic ratio, Δ is the diffusion time, and *D* is the diffusion coefficient.

#### 4.1.6 TEM

Transmission electron microscopy (TEM) was used to evaluate the lateral dimensions (*xy*) of CDs-NH_2_. Briefly, a copper grid coated with an ultrathin carbon film (01824, TED PELLA, INC.) was pretreated under a UV lamp for 30 min to generate a hydrophilic surface. Then, 0.35 μL of a 0.1 mg/mL HPLC-grade water solution of CDs-NH_2_ was dropped onto the grid and allowed to dry in the air. TEM images were recorded using a JEOL JEM 2100F microscope. Particle selection (35 particles from different areas) and size measurements were performed using the software ImageJ. The resulting size distributions and statistical analyses were generated with OriginLab.

#### 4.1.7 AFM

Atomic force microscopy (AFM) was employed to determine the height (*z*-dimension) of CDs-NH_2_. Briefly, the carbon dots were dissolved in HPLC-grade water at a concentration of 1 mg/mL, filtered through a 100 nm syringe filter, and subsequently diluted to a final concentration of 2 μg/mL. The resulting solution was sonicated for 5 min at room temperature. A 15 μL aliquot of this solution was then drop-cast onto a freshly cleaved mica disk with a diameter of 12 mm, previously dried under a nitrogen stream. The sample was allowed to dry slowly overnight under a fume hood. AFM measurements were carried out using a JPK bioAFM, operating with a TESPA-V2 tip with a frequency of 320 Hz and a scan rate of 1.5 Hz. The height data of CDs-NH_2_ were obtained by averaging measurements from multiple areas across different images, with a total of 135 peaks analyzed. The images were processed by using WSxM 7.0 software, and size distributions were analyzed by using OriginLab software. Additional details are provided in Supporting Information (Figure S8).

#### 4.1.8 STEM, EDS, and Diffraction Analysis

For STEM, EDS, and diffraction analyses, samples were prepared by dissolving CDs-NH_2_ at a concentration of 10^–4^ mg/mL in methanol. The solutions were sonicated at 25 °C for 10 min to ensure dispersion. A single drop of the solution was then deposited onto a copper grid with an ultrathin carbon film Formvar on 3 mm 400 mesh Cu grids. The grid was placed in an oven for 30 s to allow the solvent to evaporate rapidly. Subsequently, the samples were analyzed under high vacuum conditions at an accelerating voltage of 80 kV (to minimize damage and artifacts that could arise during observation), and the particles were characterized and measured in terms of morphology, shape and dimensions. STEM images were obtained using a TEM JEOL F-200 equipped with a GATAN Rio16 CMOS camera and a JEOL EDX system featuring two large-area silicon drift detectors (SDD). The analysis was performed using a beam voltage of 200 kV and an emission current of 129.4 μA. A condenser lens (CL) diaphragm with a 100 μm aperture was used during the acquisition, operating in STEM mode. For image acquisition, dark field (DF) mode was selected due to its enhanced signal quality. The STEM images were slightly modified with digital micrograph (DM) software, mainly to improve contrast relative to the Formvar/carbon support film.

EDS measurements were performed to determine the elemental composition of the nanoparticles. Spectra were collected from selected areas of the grid, and elements were identified based on their characteristic X-ray peaks. Elemental quantification was carried out using a JEOL Analysis Station 3.8. Finally, electron diffraction analysis was performed to investigate the *core* structure of the nanoparticles, distinguishing whether they were amorphous or graphitic. Images were acquired by using a camera length of 1500 mm. The pattern was processed using the CrysTBox software and the reference CIF file, in which the structure of graphite is modeled.

#### 4.1.9 X-ray Diffraction (XRD) and Raman Analysis

X-ray powder diffraction in a Bragg–Brentano geometry was performed on the CDs-NH_2_ powder using a Rigaku Miniflex diffractometer equipped with a copper tube (1.54 Å). The scans were performed in the 2Θ range of 10° to 60°, with a voltage of 30 kV, a current of 15 mA, and a scan rate of 0.5°/min.

Raman spectra were recorded using a Renishaw inVia Raman confocal microscope using a green (532 nm, 50 mW) and a red (633 nm, 17.5 mW) laser. Raman spectra were collected using a 100× objective lens over the Raman shift range of 600 to 2300 cm^–1^ from different regions of the sample. Nine regions were analyzed using both green and red lasers to ensure statistical relevance. D_3_ and D_4_ bands were identified through a four-Gaussian peak fitting as described by Shimodaira and Masui;^82^ see Figure S12. *I*(D)/*I*(G) ratio was calculated according to the three-stage model.^83^ Data fitting was performed on normalized spectra after background subtraction using SpectraGryph software (version 1.2). Both data sets were plotted and processed using OriginPro 2018, with a Savitzky–Golay smoothing of 10 points applied to the XRD data.

### 4.2 Biointeraction and Uptake of CDs-NH_2_ in Plants and Fungal Models

#### 4.2.1 Visualization of CDs-NH_2_ Uptake by Fluorescence Microscopy

CDs-NH_2_ were tested for their ability to penetrate plant and fungal cells by exploiting their intrinsic fluorescence property. The confocal analyses were performed on an Imager M2 microscope (Zeiss, Germany) equipped with a Zeiss LSM 900 spectral confocal laser scanning unit (Zeiss, Germany). For CDs-NH_2_ detection, a 488 nm, 10 mW solid laser with 490–533 nm detection bandpass was employed. Image pixel resolution was set at 1465 × 1465 and used pixel dwell time of 7.85 μs and double-way scanning. Average filtering of 2 was used during image acquisitions to improve signal-to-noise ratio of the acquired images. Images were acquired using a 40× objective lens (W Plan Apochromat 0.95 NA, Zeiss), as a Z-stack of 15 slices as average (approximately 8 μm thickness) and merged into a single image using ZEN 3.1 (Zeiss) software as maximum projection. Due to their small diameter and low autofluorescence, subsequent analyses were performed on the roots of *in vitro* cultured *A. thaliana* and *O. sativa* grown from surface-sterilized seeds.^84^ *In vitro* cultures of *B. cinerea* were used to investigate CDs-NH_2_ uptake into fungal cells. Both seedlings and fungal cells were mounted on a slide in 200 μL of 50% glycerol supplemented with an aqueous suspension of CDs-NH_2_ at a final concentration of 0.5 mg/mL. Coverslips were then placed over the samples, and fluorescence analyses were performed. The cellular uptake mechanism was investigated through colocalization analysis between CDs-NH_2_ and FM4-64 probe [*N*-(3-triethylammoniumpropyl)-4-(6-(4-(diethylamino)­phenyl)­hexatrienyl)­pyridinium dibromide] (Invitrogen, Darmstadt, Germany), according to the protocol described by Palocci et al. (2017).^85^ Briefly, *B. cinerea* hyphae were co-incubated with CDs-NH_2_ and 10 μM FM4-64 at room temperature for at least 10 min. To quantify colocalization, Pearson and Manders correlation coefficients were determined by using the confocal microscopy settings described above.

#### 4.2.2 Quantification of CDs-NH_2_ Root Uptake

Seedlings of various species, including tomato, *Arabidopsis*, and rice, were subjected to preliminary tests. The subsequent analyses focused on rice seedlings, as these have a more extensive root system and greater tolerance to *ex vitro* conditions. Seedlings obtained by *in vitro* germination of surface-sterilized seeds on half-strength Murashige and Skoog^86^ medium (MS/2) (Duchefa Biochemie, The Netherlands) were harvested from Phytatray II boxes (Sigma-Aldrich, Milan, Italy). Their root system was carefully washed to remove agar residues, and they were transferred to 200 mL MS/2 medium. Only the roots were immersed in the liquid medium, while the aboveground organs were exposed to the atmosphere. A 1 L beaker was placed over the seedlings to ensure high relative humidity. After 72 h, the seedlings were transferred to 200 mL of sterile deionized water to which CDs-NH_2_ were added at a final concentration of 40 μg/mL. The solutions were shielded from daylight to prevent them from photodegradation. At different time points after incubation (*t*_0_ = 0; *t*_1_ = 1 h; *t*_2_ = 2 h; *t*_4_ = 4 h; *t*_on_ = overnight), aliquots of the suspension were taken and stored at −20 °C to await quantification of CDs-NH_2_ by spectrofluorimetric analysis.

The uptake of CDs-NH_2_ by rice seedlings was quantified by measuring the fluorescence emission intensity of the two sample suspensions at various time points after incubation, with reference to three blank solutions (without seedlings), following the establishment of a calibration curve (Figure S13). Therefore, a stock aqueous solution of CDs-NH_2_ at a concentration of 200 μg/mL was prepared, and it was subsequently diluted to achieve final concentrations of 25, 20, 15, 10, and 5 μg/mL, respectively. The emission spectra at 450 nm of these solutions were then recorded, and the fluorescence intensity at 525 nm (the emission maximum) was measured to establish a calibration curve. The procedure was repeated five times, and the fluorescence intensity was plotted against the concentration. Subsequently, the fluorescence of both the real and the blank samples was analyzed to evaluate the content of CDs-NH_2_. All samples were previously diluted in a 1:4 ratio, after which their emission spectra at 450 nm were recorded. Using the previously recorded calibration curve, the estimated amount of absorbed and internalized CDs-NH_2_ was calculated by comparing the concentration of nanoparticles in the samples with the one of blank solutions according to eq 4:

4 $$\%{\mathrm{CDs‐NH}}_{2}^{\mathrm{absorbed}}=\left(1-\frac{C_{\mathrm{SAMPLE}}}{C_{\mathrm{BLANK}}}\right)\times 100$$

Equation 4 is the calculation of the amount of CDs-NH_2_ absorbed by rice seedlings, expressed as a percentage. *C*_SAMPLE_ and *C*_BLANK_ represent the concentrations of CDs-NH_2_ in the sample and blank solutions, respectively, as determined from the calibration curve.

#### 4.2.3 Impact of CDs-NH_2_ on Seed Germination and Seedling Development

Seed germination and seedling growth experiments were conducted in a grow chamber under a 12 h photoperiod using 90 mm diameter Petri dishes. Prior to the experiment, tomato seeds were surface sterilized by immersion in 70% ethanol for 5 min, followed by immersion in 6% sodium hypochlorite for 30 min. The seeds were then rinsed four times with sterile deionized water. Following the procedure described by Kou et al.,^51^ seeds were immersed overnight in half-strength Murashige and Skoog (MS/2) liquid medium supplemented with 0 (control), 0.033, 0.066, or 0.132 mg/mL CDs-NH_2_, and agitated at 100 rpm at 25 ± 1 °C. Three independent experiments were performed for each concentration, with 100 seeds per replicate. Seed germination was determined by the visible emergence of the radicle from the seed coat. The germination percentage was calculated using eq 5:

5 $$\mathrm{Percentage of seed germination}=\frac{\mathrm{Number of seeds germinated}}{\mathrm{Total number of seeds}}\times 100$$

Equation 5 is the formula used to calculate the percentage of seed germination, where the number of germinated seeds is expressed as a percentage of the total number of seeds.

Eleven days after treatment began, seedlings (*n* ≥ 100 per treatment) were harvested and partitioned into roots and shoots. The plant material was oven-dried at 70 °C to a constant weight, and the dry biomass was determined gravimetrically to assess growth responses.

#### 4.2.4 Analysis of the Stability of CDs-NH_2_ in Abiotic and Biological Environments

The stability of CDs-NH_2_ was evaluated over 1 week of thermal treatment. The experiments were carried out by transferring the sample solutions into a 24-well plate and storing them in a sterile incubator at 37 °C for 1 week. Fluorescence analyses were performed on CDs-NH_2_ dissolved in both Milli-Q water and in PBS at a concentration of 400 μg/mL. CDs-NH_2_ was not completely soluble in PBS; therefore, the suspension was strongly mixed before any sampling. Every day, aliquots of 50 μL were collected and stored at −20 °C before fluorescence spectroscopy analysis. For FTIR, NMR and TGA analysis, a 15 mg/mL solution of CDs-NH_2_ in Milli-Q water was prepared and stored similarly to what was described before. After 7 days, CDs were lyophilized and analyzed. For fluorescence measurements, each aliquot was diluted 1:10 to reach a final concentration of 40 μg/mL. Fluorescence emission centered at 525 nm was then recorded upon excitation at 450 nm using a FS5 spectrofluorometer (Edinburgh Instruments). Solutions were measured in quartz cuvettes from Hellma (10 mm path length, 0.5 mL). All the experiments were carried out at room temperature. Data analysis was performed by plotting both the fluorescence intensity and the normalized fluorescence intensity, the latter obtained by normalizing the values to the fluorescence recorded at time zero.

FTIR-ATR (Fourier transformation attenuated total reflection) spectra were recorded using an Invenio-X FTIR spectrometer Invenio-X (Bruker). Dried samples were analyzed between 4000–400 cm^–1^ range, acquiring 64 scans for each spectrum with 4 cm^–1^ resolution. ^1^H NMR spectrum was acquired using a Bruker AVANCE III NMR spectrometer (500 MHz). CDs-NH_2_ were dissolved in D_2_O at 10 mg/mL. For ^1^H NMR, 64 scans were recorded. The spectra were processed by using MestReNova. TGA experiments were performed under nitrogen atmosphere (25 mL min^–1^ flow rate) using a TGA Discovery (TA Instruments) on freshly lyophilized nanoparticles. The procedure consisted of a thermal ramp ranging from 30 to 700 °C at 20 °C min^–1^ heating scan. All of these spectra are represented in Figure S15.

### 4.3 Antioxidant, Antifungal, and Antibacterial Activity of CDs-NH_2_

#### 4.3.1 Materials

For antifungal assays, Potato Dextrose Agar (PDA), RPMI medium, dimethyl sulfoxide (DMSO), and Crystal Violet were obtained from Merck Life Science S.r.l. (Milan, Italy). For antioxidant and antibacterial assays, all of the reagents and solvents were purchased from Sigma-Aldrich.

#### 4.3.2 Antioxidant Tests

The antioxidant activity of CDs-NH_2_ was evaluated *in vitro* using DPPH (2,2-diphenyl-1-picrylhydrazyl) and ABTS (2,2′-azino-bis­(3-ethylbenzo­thiazoline-6-sulfonic acid)) radical assays. Both assays were performed in a 96-well microplate using a BioTek Cytation 5 cell imaging multimode reader, recording absorbance every 30 min at 520 nm (DPPH) and 734 nm (ABTS).

Aqueous solutions of CDs-NH_2_ at concentrations of 0.156, 0.312, 0.625, 1.25, and 2.50 mg/mL and a 4 mM DPPH solution in methanol were freshly prepared immediately before the experiment. In each test well, 10 μL of the methanolic DPPH solution, 80 μL of pure methanol, and 10 μL of the CDs-NH_2_ solutions were sequentially added. Three parallel control solutions were prepared:

- Positive control (PC): 10 μL of DPPH solution, 80 μL of methanol, and 10 μL of Milli-Q water.
- Negative control (NC): 90 μL of methanol and 10 μL of the CDs-NH_2_ solutions.
- Solvent blank (SB): same methanol/water mixture used in the assay to correct for background absorbance.

The ABTS radical solution was prepared by dissolving ABTS in mQ water at 7 mM and mixing it with an equal volume of a 2.45 mM ammonium persulfate aqueous solution. The solution was kept in the dark for 24 h, during which it changed from light sky blue to dark blue, indicating ABTS radical formation. CDs-NH_2_ solutions were prepared at concentrations of 16.8, 33.6, 67.2, 134.4, and 268.8 μg/mL. In each test well, 7 μL of the ABTS solution and 93 μL of the CDs-NH_2_ solutions were sequentially added. Three control solutions were prepared:

- Positive control (PC): 7 μL of ABTS and 93 μL of mQ water.
- Negative control (NC): 7 μL of mQ water and 93 μL of sample solutions.
- Solvent blank (SB): 100 μL of mQ water.

All measurements were performed in at least triplicate. Under these conditions, the tested concentrations of CDs-NH_2_ in all experiments were 15.6, 31.2, 62.5, 125, and 250 μg/mL. The antioxidant activity was calculated by using eq 6:

6 $$\mathrm{RSA}\left(\%\right)=\frac{\left({\mathrm{Abs}}_{\mathrm{PC}}-{\mathrm{Abs}}_{\mathrm{SB}}\right)-\left({\mathrm{Abs}}_{\mathrm{Sample}}-{\mathrm{Abs}}_{\mathrm{NC}}-{\mathrm{Abs}}_{\mathrm{SB}}\right)}{\left({\mathrm{Abs}}_{\mathrm{PC}}-{\mathrm{Abs}}_{\mathrm{SB}}\right)}\times 100$$

Equation 6 is the formula showing the calculation of the radical scavenging activity (RSA) of CDs-NH_2_, expressed as percentage, using the absorbances of the sample and blank solutions as illustrated above.

The radical scavenging activity of CDs-NH_2_ was quantified in terms of the half-maximal inhibitory concentration (IC_50_), defined as the concentration required to reduce 50% of the initial radical signal in each assay.

#### 4.3.3 Antifungal Susceptibility Test against *B. cinerea*

The antifungal activity of CDs-NH_2_ was evaluated using the broth microdilution method, following the standardized mold susceptibility testing protocol^87^ with some modifications. For this study, *B. cinerea* DSM 877 was sourced from the DSMZ (Leibniz Institute, DSMZ-German Collection of Microorganisms and Cell Cultures GmbH). The inoculum was suspended in RPMI-1640 broth and adjusted to a concentration between 0.4 × 10^4^ and 4 × 10^4^ conidia/mL. CDs-NH_2_ were diluted in RPMI-1640 medium buffered with MOPS (4-morpholine­propanesulfonic acid) to maintain a stable pH. The tested concentrations ranged from 500 to 0.98 μg/mL, prepared through serial dilutions in 96-well microplates. Minimal inhibitory concentrations (MICs) were determined by comparing fungal growth in treated wells to that in untreated controls. The readings were taken after 72 h of incubation at 23 °C. Each experiment was conducted in duplicate and repeated at least three times to ensure reproducibility.

#### 4.3.4 Activity against *B. cinerea* Biofilm

The biofilm formation assay was performed in flat-bottomed 48-well microtiter plates, following previously established protocols with minor modifications.^34^ 200 μL of *B. cinerea* DSM 877 conidia in RPMI-1640 medium buffered with MOPS, were added into each well along with 200 μL of CDs-NH_2_ solutions at concentrations ranging from 500 μg/mL to 31.25 μg/mL. The plates were incubated at 23 °C for 72 h to facilitate biofilm development. After incubation, the medium was discarded, and nonadherent cells were washed away using PBS. The biomass of the biofilm was quantified via the crystal violet (CV) assay. Each experiment was conducted in triplicate and repeated at least three times to ensure reproducibility.

#### 4.3.5 Bacterial Growth Inhibition

The antibacterial activity of CDs-NH_2_ at different concentrations was tested against the model plant pathogen *P. syringae* pv. *tomato* DC3000 (PtoDC3000) by using the agar plate well-diffusion method previously described by other authors.^88^ The bacterial strain was grown on King’s B medium plates at 27 °C for 48 h. 50 μL of the bacterial suspension (OD_600_ = 0.5) was spread onto three plates, corresponding to three replicates, covering the entire surface. Five equidistant wells of approximately 0.6 mm diameter were cut on the plates using the back of sterile 20–200 μL pipet tips. The wells were filled with 80 μL of CDs-NH_2_ suspensions at the following concentrations: 0.25, 0.50, 1.0, 5, and 10 mg/mL. The plates were put on 27 °C for 48 h. The diameter of the inhibition zone at different concentrations of CDs-NH_2_ was measured.

#### 4.3.6 Bacterial Antibiofilm Activity Assay

Bacterial biofilm production by *P. syringae* pv. *tomato* was assessed following the protocol described by Shao et al.^89^ with few adjustments. Briefly, the suspensions of CDs-NH_2_ in Luria–Bertani (LB) medium at different concentrations (thesis at 0.25, 0.50, 1.0, 2.0, 2.5, and 3.0 mg/mL) were inoculated with a PtoDC3000 suspension reaching the final concentration of 10^6^ CFU/mL and incubated at 27 °C without shaking. After 24 h, the cultures were gently discarded, and the tubes were washed three times with 1 mL of sterile deionized water. Then, 1.5 mL of 0.1% crystal violet were added to the tubes and let the bacterial biofilm stain for 20 min without shaking. The dye was discarded, and the tubes were rinsed three times in water and allowed to dry overnight. The biofilm was eluted with 1.5 mL of 100% ethanol, and the tubes were shaken for 20 min to ensure that the dye had dissolved completely. The eluted samples were measured at OD_590 nm_ using a spectrophotometer. Six replicates for each thesis were considered, and the experiment was repeated twice. Six replicates of bacterial suspension on the LB medium were used as a control.

#### 4.3.7 In Planta Assay: Effect of CDs-NH_2_ on Bacterial Speck of Tomato

Tomato plants were grown in seedling trays for 2 weeks and then transplanted into larger pots. Plants were grown under 16 h days at 22 °C and each plant sprayed with approximately 5 mL of CDs-NH_2_ at 200 μg/mL when 3 to 4 weeks old and sprayed again 2 days before the bacterial inoculation to prime the plants to induce their basal plant defense. To evaluate the possible influence of CDs-NH_2_ treatments on disease severity, 20 plants were treated with CDs-NH_2_ suspension as described above and then inoculated with PtoDC3000 and 20 plants were only inoculated with the bacteria without having previously received the CDs-NH_2_ treatment. For the inoculum, PtoDC3000 was streaked from glycerol stock onto King’s broth plates and grown at 27 °C for 48 h. Then the bacterium was restreaked onto new plates and grown for 24 h. The bacterium was scraped off the plates, resuspended, and diluted in sterile H_2_O at the final bacterial concentration of 10^8^ CFU/mL for infection. The tomato plants, 24 h before being inoculated, were placed into plastic bags and watered with deionized water to ensure high humidity, then were inoculated by spraying the bacterial suspension prepared as described above. Twenty-four hours after inoculation, the plastic bags were carefully removed. As controls, three plants were sprayed only with H_2_O and three plants only with CDs-NH_2_. Disease symptoms were evaluated 7 days post inoculation and scored on a disease severity scale as (0), no symptoms; (1) few water-soaked lesions with halo; (2) pinpoint lesions of <1 mm surrounded by yellow halo; (3) evident necrotic dots of >1 mm, chlorosis and coalescence area. Severity rate was determined by the incidence data, expressed as the sum of the number of plants affected by each category multiplied by their grade and divided by the total number of assessed plants.

### 4.4 Metabolomics

#### 4.4.1 Materials

Deuterium oxide (D_2_O, 99.86% D), deuterated chloroform (CDCl_3_, 99.8% D), 3-(trimethylsilyl)­propionic-2,2,3,3-*d*_4_ acid sodium salt (TSP, 99% D), hexamethyldisiloxane (HMDSO), chloroform (CHCl_3_), and methanol (CH_3_OH) were purchased from Sigma (Sigma-Aldrich, Milano, Italy). Standard 5 mm NMR tubes and sealing caps were purchased from Hilgenberg (Hilgenberg GmbH, Malsfeld, Germany).

#### 4.4.2 Extraction Protocol

Tomato leaves for each sample grouping (H_2_O, CDs-NH_2_, Pto, CDs-NH_2_+Pto) were collected and stored at −80 °C. The extraction was carried out by a modified Bligh–Dryer protocol,^69^ weighing approximately 0.500 g of leaves for each sample. Briefly, the extraction consisted of grounding in a mortar under liquid nitrogen the tomato leaves and then transferring them into a test tube with 2 mL of cold methanol. After this step, 2 mL of cold chloroform and 1 mL of distilled water were added, stirring the tube after each solvent addition. The samples were then stored at 4 °C for 12 h and, following this, centrifuged for 30 min at 11 000 rpm at 4 °C. The upper hydrophilic phase and lower lipophilic phase were then separated and dried under nitrogen flow. The hydrophilic phase was resuspended in 0.7 mL of D_2_O containing 3-(trimethylsilyl)­propionic-2,2,3,3-*d*_4_ acid sodium salt (TSP, 2 mM), while the lipophilic phase was resuspended in 0.7 mL of CDCl_3_ containing hexamethyldisiloxane (HMDSO, 2 mM), as an internal and chemical shift reference standard, both later analyzed by ^1^H NMR.

#### 4.4.3 NMR-Based Metabolomic Analysis

The NMR experiments were carried out at 298 K on a JNM-ECZ 600R spectrometer (JEOL Ltd., Tokyo, Japan) operating at the proton frequency of 600 MHz and equipped with a multinuclear z-gradient inverse probe head. The monodimensional ^1^H NMR experiments were carried out for quantitative analysis, employing a presaturation pulse sequence for water suppression with a time length of 2 s (hydroalcoholic samples only), a spectral width of 9.03 kHz and 64k data points, corresponding to an acquisition time of 5.81 s. The pulse length of 90° flip angle was set to 8.3 μs, and the recycling delay was set to 5.72 s.

Bidimensional ^1^H–^1^H TOCSY and ^1^H–^13^C HSQC experiments were acquired for the resonance assignment, following parameters reported in Spinelli et al.^90^ Spectral assignment was carried out based on chemical shift, multiplicity, J couplings, ^1^H–^1^H TOCSY and ^1^H–^13^C HSQC correlations, following the protocol reported by Sciubba et al.^91^ Quantities were expressed in mg/100g through comparison of the relative integrals with reference concentration and normalized to the number of protons (TSP, 9 protons; HMDSO, 18 protons) and to the starting fresh weight of the sample.

Multivariate and univariate analyses were performed on MATLAB R2023a (MathWorks, Natick, Massachusetts, USA) with the Statistics and Machine Learning Toolbox package, with a home-built script.

Univariate analysis was carried out by a one-way ANOVA test; normality and homoscedasticity of each metabolite were evaluated with Shapiro–Wilk and Brown–Forsythe tests. Depending on the results of these tests, the ANOVA test and Kruskal–Wallis were performed. Multiple groups comparison was corrected with Dunnett, choosing as control for comparison TL H_2_O labeled samples.

Prior to multivariate analysis, the matrix was treated by mean centering and autoscaled to equalize the importance of the variation of each variable. Following this, principal component analysis was done to observe multivariate metabolite trends among different groups of samples.

## Supplementary Material





## Notes

Additional data underlying this study, including the Kaiser test, ^19^F NMR, DOSY, STEM AFM, EDS, electron diffraction, Raman spectroscopy, fluorescence calibration curves, stability experiments, antioxidant activity studies, antibacterial results, time lapse acquisition of CDs-NH_2_ internalization in *B. cinerea* cells, and metabolomic analyses (PDF)
Video S1 of fluorescence in fungal cells (MP4)
