# Supplementary material for: Bioactive Amino-Carbon Dots for Sustainable Crop Protection: Cellular Uptake and Metabolomic Insights into the Antifungal and Antibacterial Activity in Tomato Plants
Source: ACS Appl Mater Interfaces. 2026 Apr 30;18(18):25873–91. doi: 10.1021/acsami.6c03466 (PMC13181715; doi:10.1021/acsami.6c03466)
Supplement: Supplementary file 1 [file am6c03466_si_001.pdf]

Supporting Information

Bioactive Amino-Carbon Dots for Sustainable Crop Protection: Cellular Uptake and Metabolomic Insights into the Antifungal and Antibacterial Activity in Tomato Plants

Alessandro Camilli,[1] Adriano Patriarca,[1] Patrizia Ferrante,[2] Elisa Brasili,[3,4] Pierfrancesco Atanasio,[5] Corrado Di Conzo,[6] Elisa Sturabotti,[7] Laura Verdolini,[8] Simone D'Angeli,[3] Marco Rossi,[5,9] Fabrizio Vetica,[1] Francesca Leonelli,[1] Giovanna Simonetti,[3]* Alessio Valletta [3]**

[1] Department of Chemistry, Sapienza University of Rome, Piazzale Aldo Moro 5, 00185 Rome, Italy

[2] Consiglio per la ricerca in agricoltura e l'analisi dell'economia agraria (CREA) – Centro di Olivicoltura, Frutticoltura e Agrumicoltura (CREA-OFA), Via di Fioranello, 52; 00134 Roma, Italy

[3] Department of Environmental Biology, Sapienza University of Rome, Piazzale Aldo Moro 5, 00185 Rome, Italy

[4] NMR-Based Metabolomics Laboratory, University of Rome Sapienza, Piazzale Aldo Moro 5, 00185 Rome, Italy

[5] Department of Basic and Applied Sciences for Engineering (SBAI), Sapienza University of Rome, Via A. Scarpa 14, 00161 Rome, Italy

[6] Department of Applied Science and Technology (DISAT), Polytechnic of Turin, Corso Castelfilardo 39, 10129 Torino, Italy

[7] Center for Cooperative Research in Biomaterials (CIC biomaGUNE), Basque Research and Technology Alliance (BRTA), Donostia-San Sebastián 20014, Spain

[8] Department of Public Health and Infectious Diseases, Sapienza University of Rome, Piazzale Aldo Moro 5, 00185 Rome, Italy

[9] Research Centre for Nanotechnology applied to Engineering of Sapienza University of Rome (CNIS), Piazzale A. Moro 5, 00185 Rome, Italy

Corresponding Authors

*Francesca Leonelli – Department of Chemistry, Sapienza University of Rome, Piazzale Aldo Moro 5, 00185 Rome, Italy.

orcid.org/0000-0002-4135-5727

Email: francesca.leonelli@uniroma1.it

*Giovanna Simonetti – Department of Environmental Biology, Sapienza University of Rome, Piazzale Aldo Moro 5, 00185 Rome, Italy.

orcid.org/0000-0003-4321-4626

Email: giovanna.simonetti@uniroma1.it

*Alessio Valletta – Department of Environmental Biology, Sapienza University of Rome, Piazzale Aldo Moro 5, 00185 Rome, Italy.

orcid.org/0000-0002-8988-1400

Email: alessio.valletta@uniroma1.it

Synthesis of CDs-NH_2. Microwave assisted solvothermal synthesis of CDs-NH_2 from glucosamine hydrochloride and *m*-diaminobenzene (**Figure S1**). The reaction was performed in an open vessel using a domestic microwave oven (800 W, 3 minutes), yielding green fluorescent CDs-NH_2.

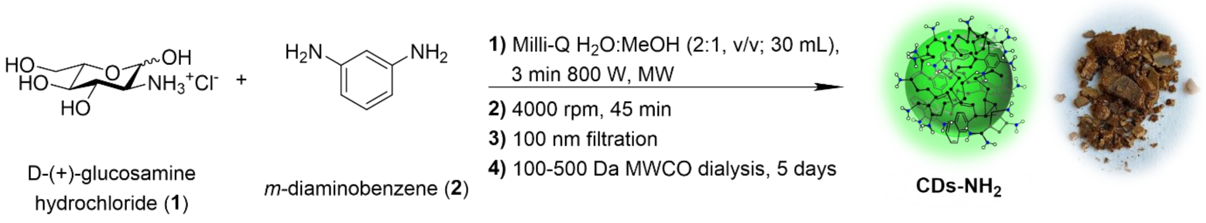

Figure S1. Schematic representation of the synthesis and purification of CDs-NH_2, and photograph of the resulting CDs-NH_2 powder. Blue and white spheres represent the nitrogen and hydrogen atoms, respectively, of the primary amino groups on the CDs-NH_2 surface.

Summary of Previously Published Characterization Data of CDs-NH_2. The following panel summarizes the physicochemical characterization of CDs-NH_2 as previously reported [1], including NMR, FTIR, absorption, fluorescence, elemental analysis and XPS (**Figures S2A, B, C, D, E, F, G, H, I**). Collectively, they show that CDs

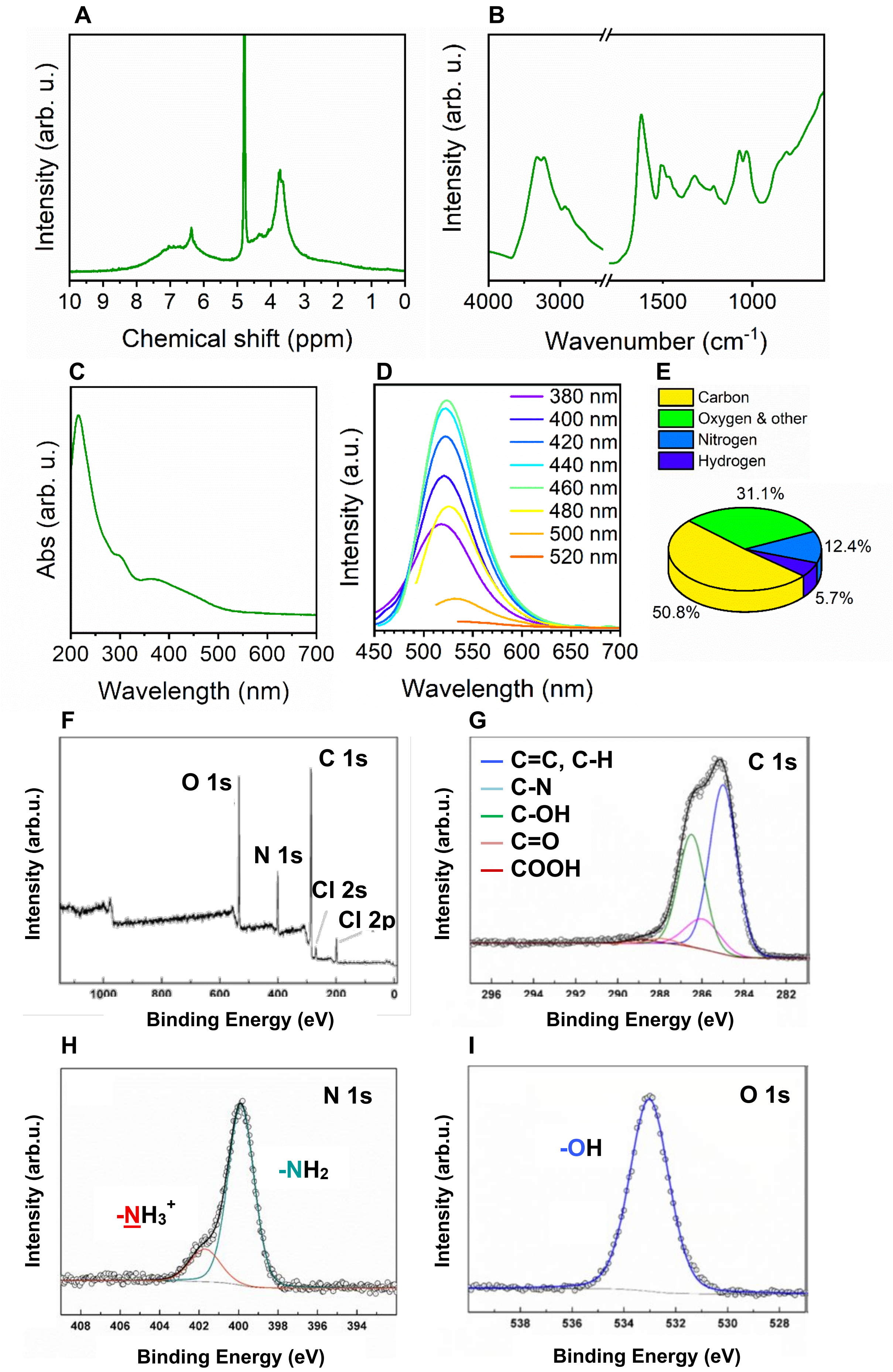
A
Intensity (arb. u.)
Chemical shift (ppm)
B
Intensity (arb. u.)
Wavenumber (cm-1)
C
Abs (arb. u.)
Wavelength (nm)
D
380 nm
400 nm
420 nm
440 nm
460 nm
480 nm
500 nm
520 nm
Intensity (a.u.)
Wavelength (nm)
E
Carbon
Oxygen & other
Nitrogen
Hydrogen
31.1%
12.4%
5.7%
50.8%
F
O 1s
C 1s
N 1s
Cl 2s
Cl 2p
Intensity (arb.u.)
Binding Energy (eV)
G
C=C, C-H
C-N
C-OH
C=O
COOH
C 1s
Intensity (arb.u.)
Binding Energy (eV)
H
N 1s
-NH2
-NH3+
Intensity (arb.u.)
Binding Energy (eV)
I
O 1s
-OH
Intensity (arb.u.)
Binding Energy (eV)

Figure S2. (A) ^1H NMR spectrum of CDs–NH_2 recorded in D_2O. (B) ATR-FTIR spectrum. (C) UV–Vis absorption spectrum. (D) Fluorescence emission spectrum. (E) Elemental analysis. (F) XPS survey spectrum and high-resolution spectra of C (G), N (H), and O (I).

Kaiser Test of CDs-NH_2. Primary amino groups of CDs-NH_2 were first estimated through Kaiser Test. **Figure S3** shows both the individual and average values obtained from the Kaiser test, which was used to quantify the content of primary amine groups in the CDs-NH_2 samples. The average amine content, calculated from six independent samples, was found to be 1.22 ± 0.05 mmol of primary amine groups per gram of CDs-NH_2, indicating a high degree of surface functionalization.

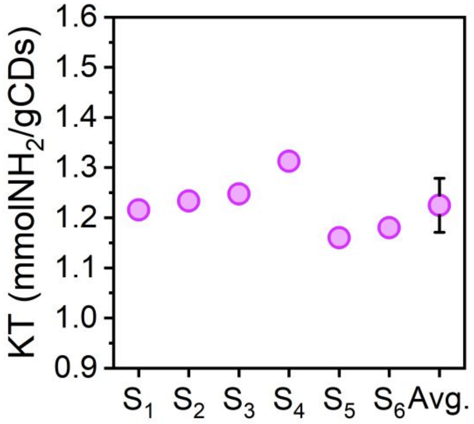

Figure S3. Graphical representation of primary amine content in CDs-NH_2 determined via the Kaiser test. Data are shown as individual values and average ± SE, calculated from six independent samples.

Quantification of Surface Primary Amines on CDs-NH_2 by ^{19}F NMR. Primary amino groups on the CDs–NH_2 surface were further quantified through functionalization with FBA. **Figure S4**

shows the ^{19}F NMR spectrum of the reaction mixture; based on the integral values and according to **Equation 2**, the amount of reactive primary amino groups was calculated to be 1.90 mmol NH_2 per g of CDs–NH_2. Since the ^{19}F NMR spectrum of the reaction mixture displayed an additional peak at –106.9 ppm, a blank experiment was performed to evaluate whether this signal originated from the aldehyde itself or from molecular impurities present in the nanoparticles. To this end, FBA was subjected to the same reaction conditions in the absence of CDs-NH_2, and its ^{19}F NMR spectrum was recorded (**Figure S5**). The spectrum displayed the same signal at –106.9 ppm, confirming that the peak originated from FBA itself.

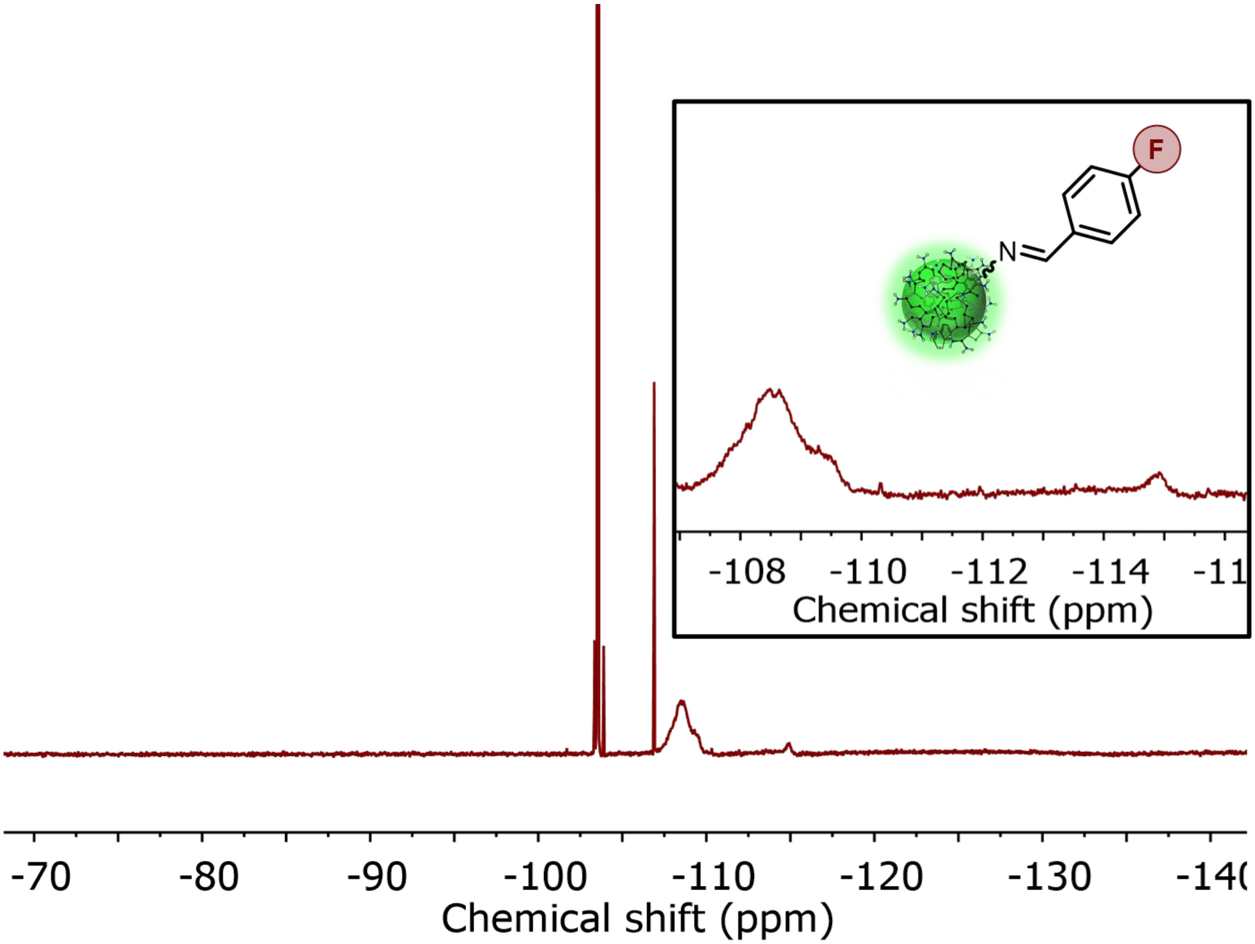

Figure S4. ^{19}F NMR spectrum of imines and iminium functionalities formed upon the functionalization of CDs-NH_2 (6.0 mg) with FBA (60 μmol) in DMSO-d_6 after 24 hours.

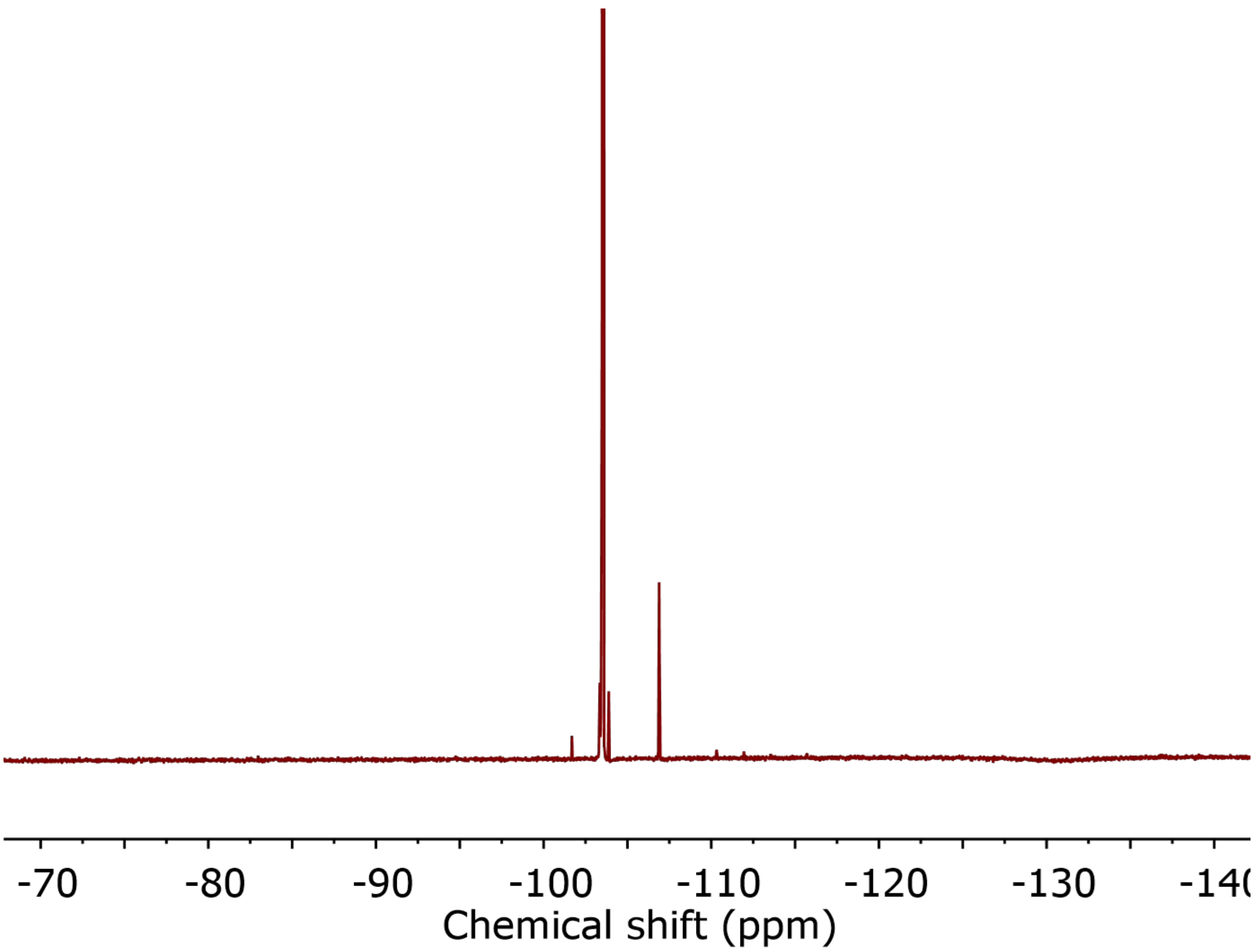

Figure S5. ^{19}F NMR spectrum of BFA in DMSO-d_6 after 24 hours. The spectrum shows that the signal centred at –106.9 ppm originates independently from the presence of CDs-NH_2.

DOSY measurements. From the elaboration of the exponential decay of the intensity of proton peaks, the diffusion decay curves were fitted assuming a monoexponential behaviour (**Figure S6**). To obtain an accurate assessment of polydispersity, the spectra were processed by dividing the peak integrals into smaller segments corresponding to aromatic and aliphatic protons, thus avoiding the use of averaged diffusion coefficient values thus avoiding the use of averaged diffusion coefficient values. The diffusion coefficients of the aromatic and aliphatic proton signals

were 1.45 ± 0.10 × 10^{-6} cm²/s and 1.64 ± 0.04 × 10^{-6} cm²/s, respectively. This difference was negligible, yielding a mean value of 1.55 ± 0.06 × 10^{-6} cm²/s, indicative of small nanoparticles diffusing as a single entity.

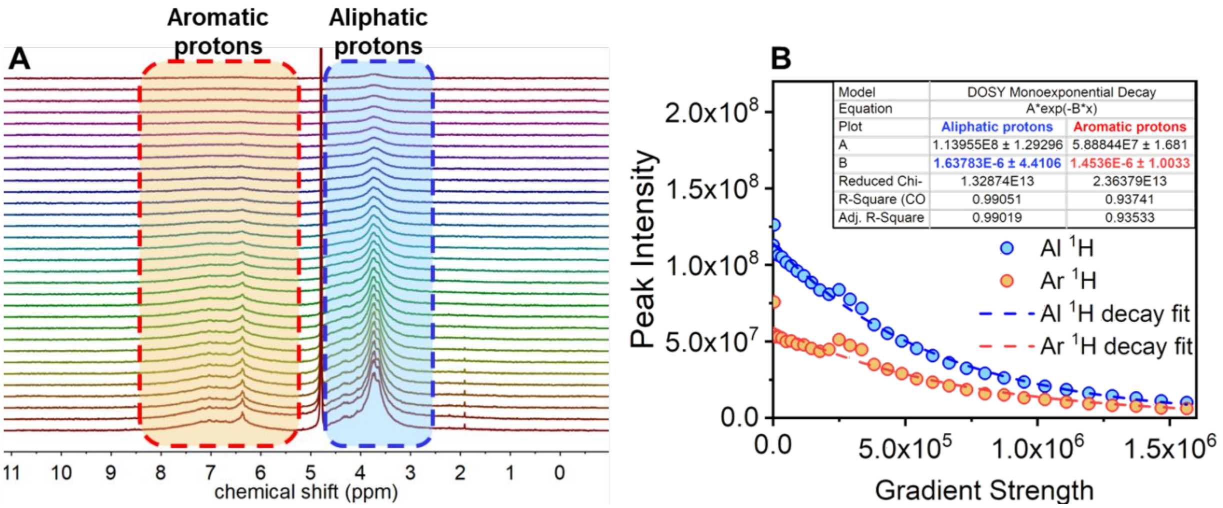

Figure S6. (A) DOSY spectrum acquired with 32 gradient points in diffusion for CDs-NH_2, calibrated using the residual water peak as reference. Aromatic protons are highlighted in red, while aliphatic protons are shown in blue. (B) Monoexponential decay fits of DOSY signals corresponding to aliphatic (blue) and aromatic (red) proton regions, showing diffusion coefficients of 1.64 ± 0.04 × 10^{-6} cm²/s and 1.45 ± 0.10 × 10^{-6} cm²/s, respectively.

TEM/STEM. Together with the TEM analysis reported in the main text, an additional TEM image acquired at a magnification of 200 nm is provided, showing multiple nanoparticles, some of which appear partially aggregated, and confirming their size distribution (**Figure S7A**). Furthermore, a complementary STEM image was acquired to provide higher contrast and enhanced visualization

of the CDs–NH_2 morphology, revealing a single and well-defined nanoparticle of approximately 3 nm (**Figure S7B, C**).

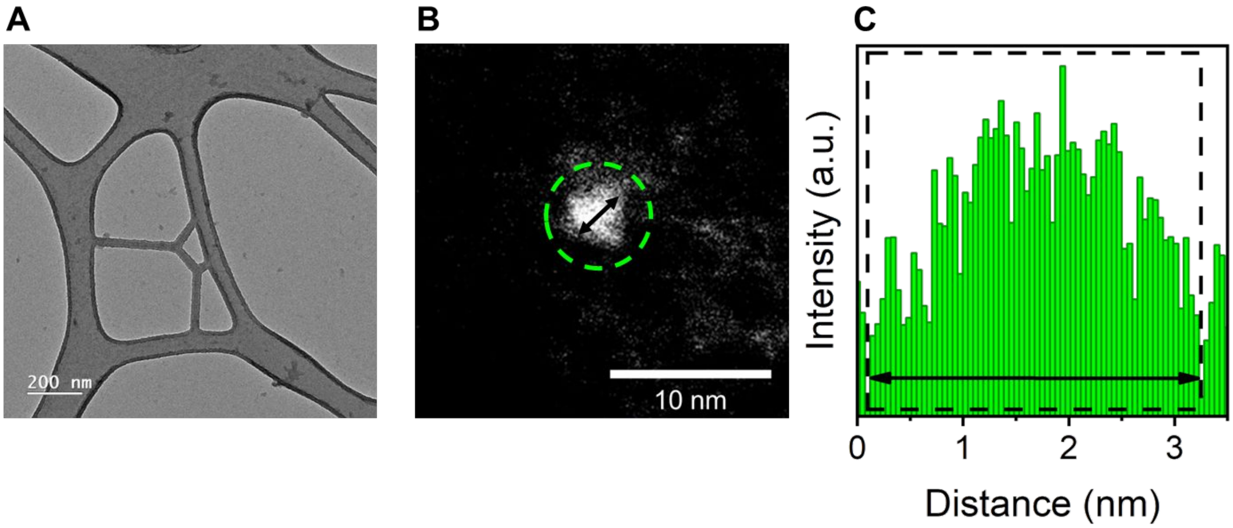

Figure S7. (A) TEM image of CDs–NH_2 at 200 nm scale, showing multiple nanoparticles with partial aggregation. (B) STEM micrograph of a single nanoparticle, with lateral dimensions of approximately 3 nm. (C) The histogram shows the actual size (3.2 nm) of the particle under examination. The relative intensity of each bin reflects the dimensional value of each pixel within the image. The pixel size value is 0.067 nm, which is also the lateral resolution limit of the microscope.

AFM. AFM measurements were conducted on CDs-NH_2 in different regions of the sample to evaluate their surface morphology and size. The resulting topographic images, together with the corresponding height profiles, were recorded at different magnifications (**Figure S8**).

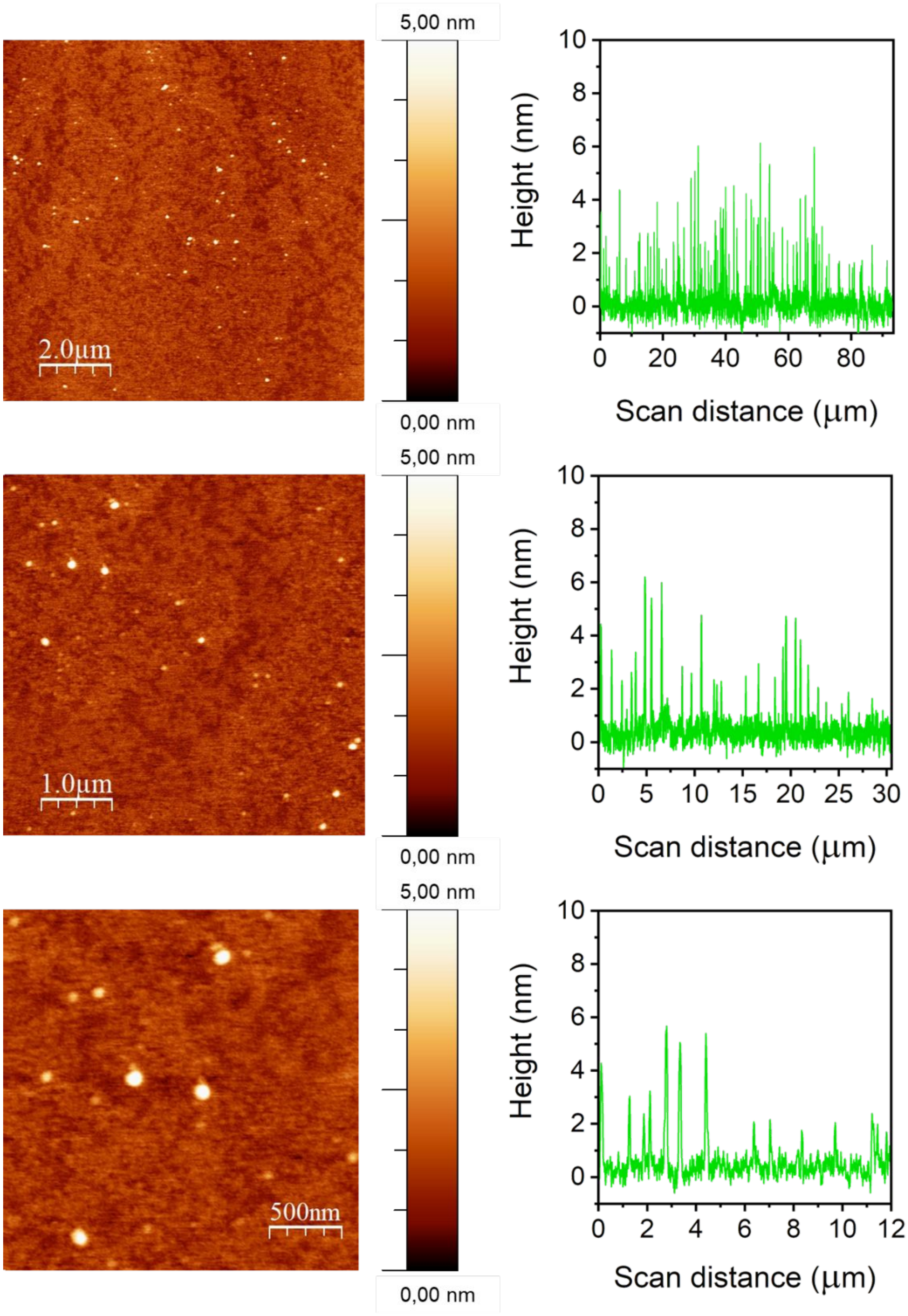
5,00 nm
0,00 nm
2.0µm
Height (nm)
Scan distance (µm)
5,00 nm
0,00 nm
1.0µm
Height (nm)
Scan distance (µm)
5,00 nm
0,00 nm
500nm
Height (nm)
Scan distance (µm)

Figure S8. AFM images of CDs-NH_2 acquired from three distinct regions of the sample, each shown at a different magnification. The corresponding height profiles are reported next to each image. Scale bars correspond to 2 μm, 1 μm, and 500 nm, respectively.

EDS analysis. EDS characterization performed at 200 keV (**Figure S9**). The EDS analysis was conducted on a larger particle than the one shown in **Figure S7B**. The reason for this choice was dictated by the fact that with smaller particles it was not possible to obtain appreciable data, as the particles disintegrated during the analysis.

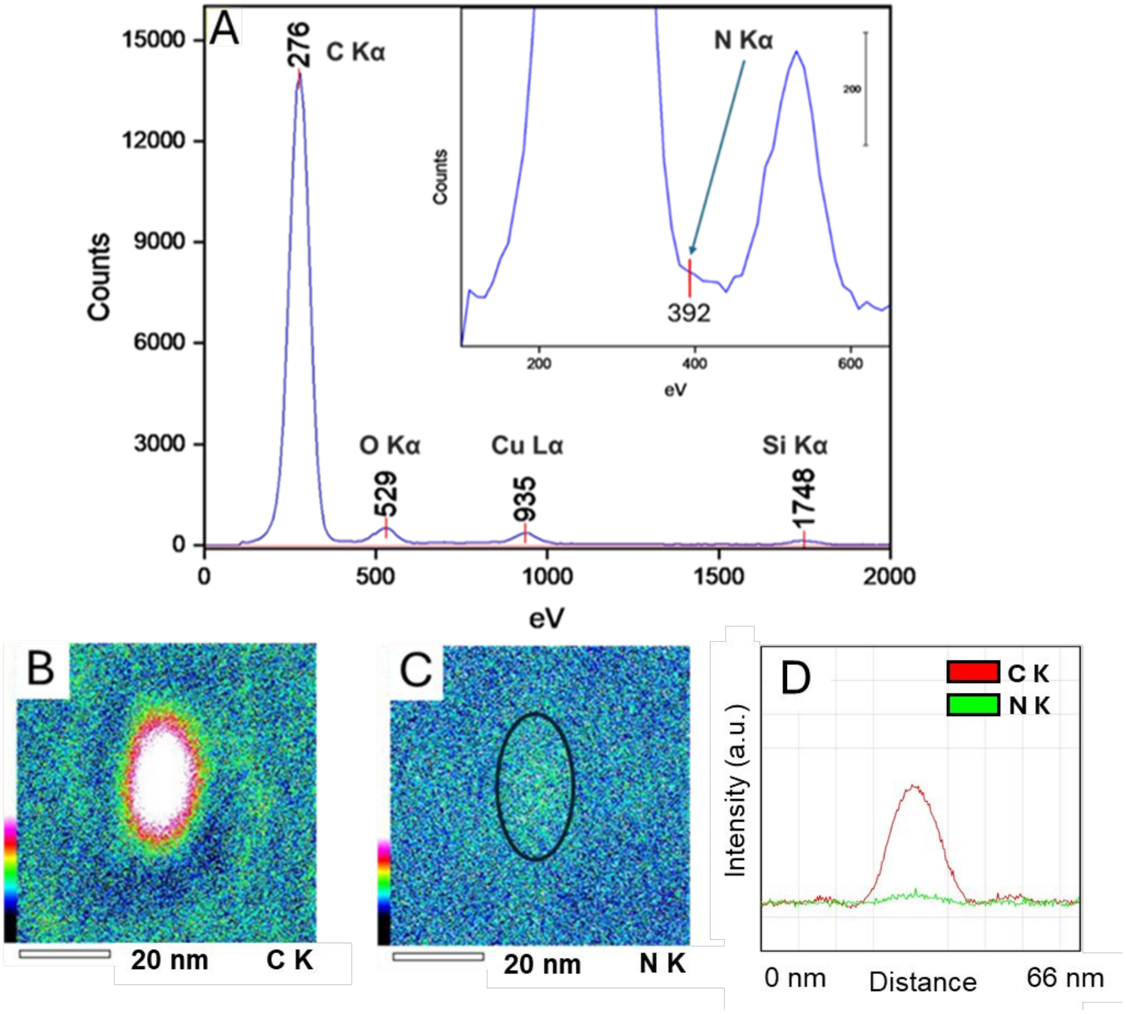

Figure S9. (A) EDS spectrum in the 0–2 keV range. (B) Elemental map based on the carbon Kα signal intensity. (C) Elemental map based on the nitrogen Kα signal intensity. (D) EDS profile of the nanoparticle under investigation, showing two distinct peaks corresponding to carbon (in red) and nitrogen (green).

Electron diffraction analysis. **Figure S10** reports the SAED pattern and corresponding diffraction intensity profile of a CDs-NH_2 particle, highlighting features related to its internal structure.

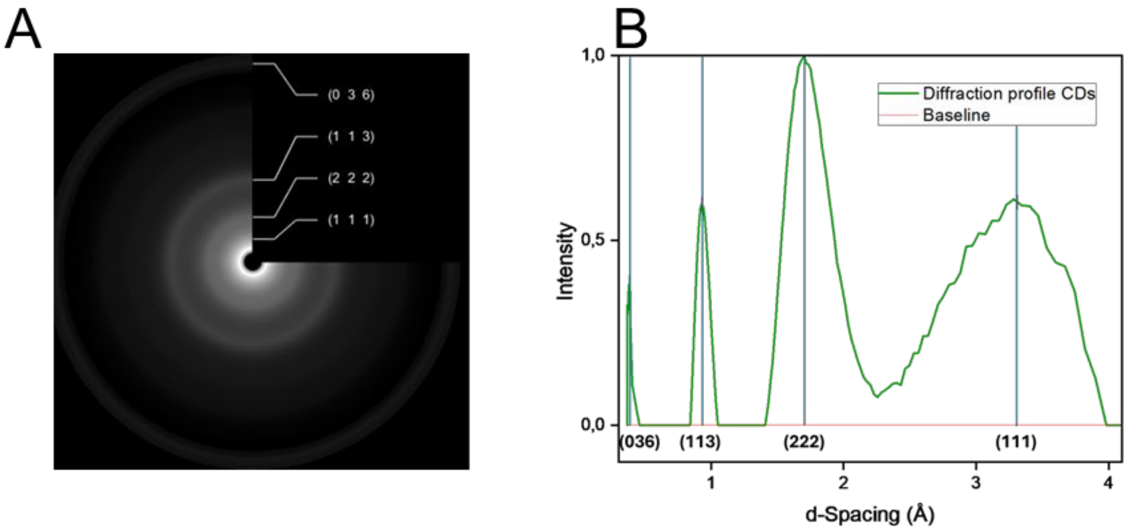

Figure S10. (A) SAED pattern of a CDs-NH_2 single particle obtained with a 60 µm aperture. (B) Diffraction profile shown as a function of d-Spacing.

Raman analysis. Table S1 shows the determined values for the D and G bands, the calculated I_D/I_G ratio, and the crystallite dimensions obtained with and without energy corrections. Specifically, the I_D/I_G ratio was found to be 0.80 ± 0.05 under green light, and 0.79 ± 0.04 under red light, respectively. The crystallite size (L_a) was estimated using the Tuinstra–Koenig relation, both

without[2] and with energy correction.[3] The former yielded an average size of 5.6 ± 0.2 nm (range: 5.0–6.6 nm), while the latter resulted in a broader distribution, ranging from 5.0 to 11.4 nm. Although these dimensions appear to diverge from those obtained through microscopic analyses, it is important to note that the average graphitic order and domain size estimated using the Tuinstra–Koenig relation should be interpreted as semi-quantitative indicators rather than absolute particle sizes.[4] These parameters were determined across nine different regions of the sample, showing comparable values. The corresponding spectra are shown in **Figure S11A, B**, while **Figure S12** illustrates the four-Gaussian peak fitting used for the deconvolution of the two main bands.

Table S1. Raman spectral parameters calculated in the different regions investigated.

Region	λ_{lamp} (nm)	D-band (cm^{-1})	G-band (cm^{-1})	I_D/I_G ratio	$^{*}L_a$ (nm)	$^{**}L_a$ (nm)
1	633	1359.5	1594.0	0.74	5.91	11.4
2	633	1358.6	1584.1	0.86	5.1	9.9
3	633	1355.3	1594.8	0.84	5.23	10.1
4	633	1362.6	1597.4	0.75	5.86	11.4
5	633	1360.2	1590.3	0.78	5.64	10.9
6	532	1363.0	1588.2	0.79	5.59	5.4
7	532	1357.5	1586.5	0.85	5.19	5.0
8	532	1365.6	1606.5	0.67	6.57	6.3
9	532	1357.1	1601.0	0.88	5.0	4.8

* L_a calculated without energy correction and ** with energy correction.

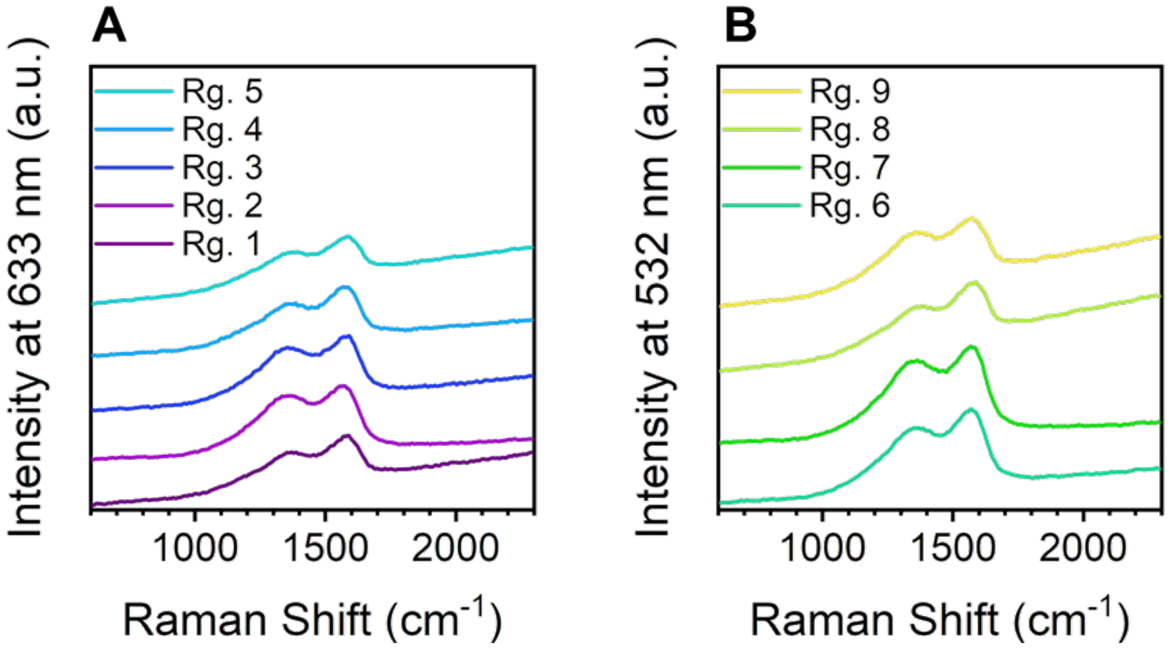

Figure S11. Raman spectra acquired from nine distinct regions of the sample using excitation wavelengths of (A) 633 nm and (B) 532 nm.

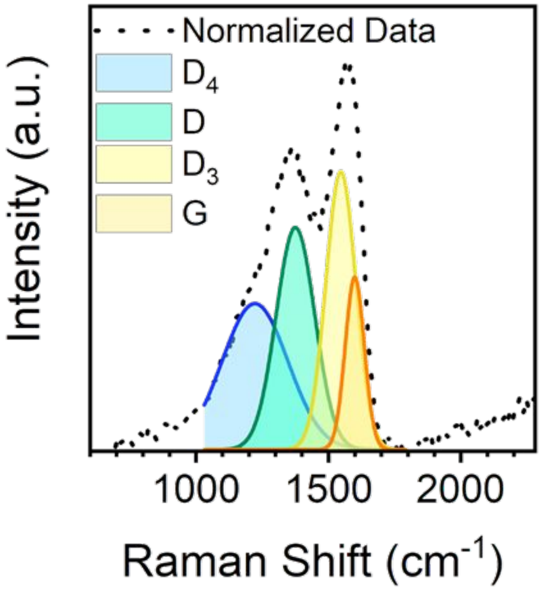

Figure S12. Deconvolution of a Raman spectrum acquired with 633 nm excitation, showing the fitted components of the D and G bands. Coloured curves represent individual Gaussian contributions, while the black dotted line indicates the overall fitted spectrum.

Uptake of $CDs\text{-}NH_2$ by *Botrytis cinerea* cells. Video S1. A time lapse acquisition of 15 frames for 14 minutes has been performed on a Z-stack of 5 slides (8 µm total thickness), repeated two times consecutively (28 minutes total time).

Fluorescence calibration curve for quantification of $CDs\text{-}NH_2$ uptake in rice seedlings. A fluorescence calibration curve was generated by measuring the emission intensity of $CDs\text{-}NH_2$ at 525 nm upon excitation at 450 nm (**Figure S13**), to quantify nanoparticle uptake by rice seedlings (**Figure S14**). The concentration range evaluated spanned from 5 to 25%µg/mL. Fluorescence data were collected from five independent replicates, averaged, and used to construct the calibration curve. The analysis was performed in quintuplicate to ensure statistical reliability. The resulting linear equation (**Equation S1**) was:

$$I = 2{,}2085 \cdot 10^5 \cdot C$$

Equation S1: Equation describing the fluorescence intensity of $CDs\text{-}NH_2$ as a function of the concentration, where I represents the fluorescence emission of the sample, C is the concentration of $CDs\text{-}NH_2$ expressed in µg/mL in the solutions, $2{,}2085 \cdot 10^5$ is the slope. The intercept was fitted to zero since blank solutions of pure water do not emit any radiation.

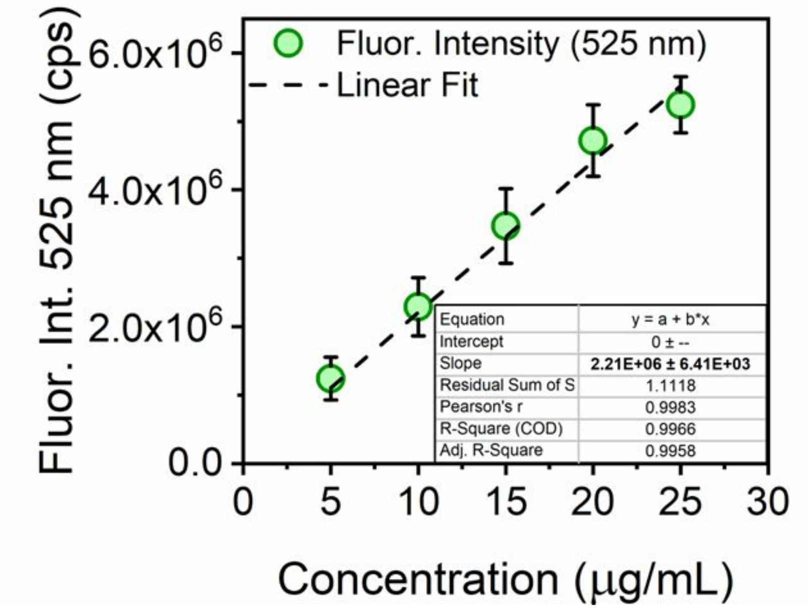

Figure S13. Calibration curve of fluorescence recorded at 525 nm for CDs-NH_2 upon excitation at 450 nm.

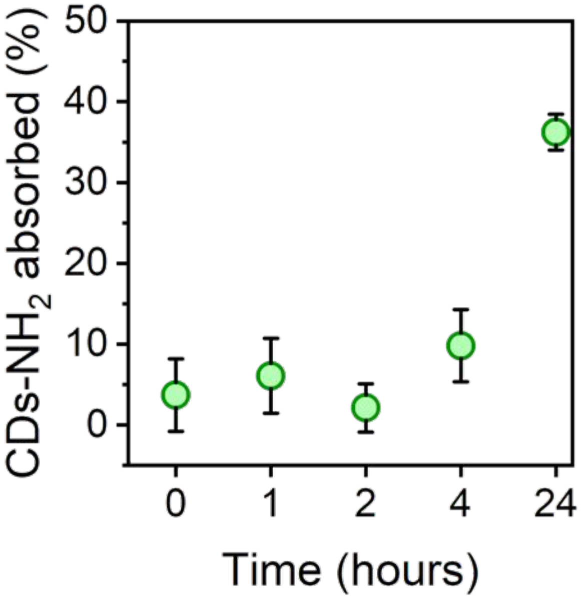

Figure S14. Percentage of CDs-NH_2 adsorbed by rice seedlings as a function of time. These data were obtained by measuring the decrease of external CDs-NH_2 concentration over the time through **Equation 4**. Each data represents the average of three independent measurements ± SE.

Measurements were performed in sterile, deionized water at room temperature with solutions excited at 450 nm and emission intensity measured at 525 nm.

Analysis of the Stability of CDs-NH_2 in abiotic and biological environments. Figure S15 presents the results obtained from incubating CDs–NH_2 in a thermostated incubator at 37 ˚C for one week. **Figures S16A** and **B** show the day-by-day evolution of the fluorescence intensity during the treatment, highlighting any fluctuations in the emission signal. **Figures S14C**, **D** and **E** report the FTIR, NMR and TGA profiles, respectively, collected at time zero and at the end of the incubation. The analysis was then extended to a biotic environment. **Figure S15** shows the bright-field and corresponding epifluorescence micrographs of Arabidopsis roots incubated with CDs-NH_2 at various time points over the same temporal window.

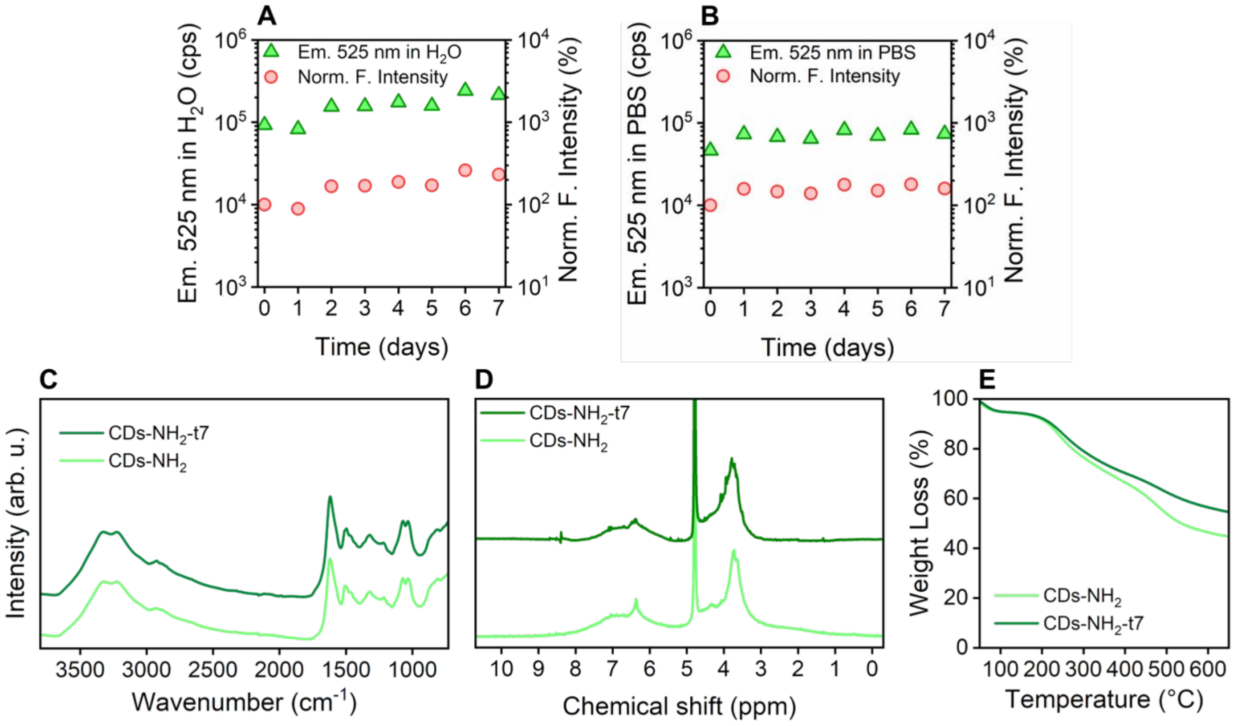

Figure S15. Spectral characterization of CDs–NH_2 before and after weekly treatment at 37 °C. (A-B) Daily fluorescence intensity at 525 nm (λ_ex = 450 nm) monitored over 7 days, showing absolute emission values (green triangles) and normalized intensities relative to time zero (red circles) in (A) Milli-Q water and (B) PBS. (C–E) FTIR (C), NMR (D), and TGA (E) spectra recorded before (pale green) and after thermal treatment (dark green).

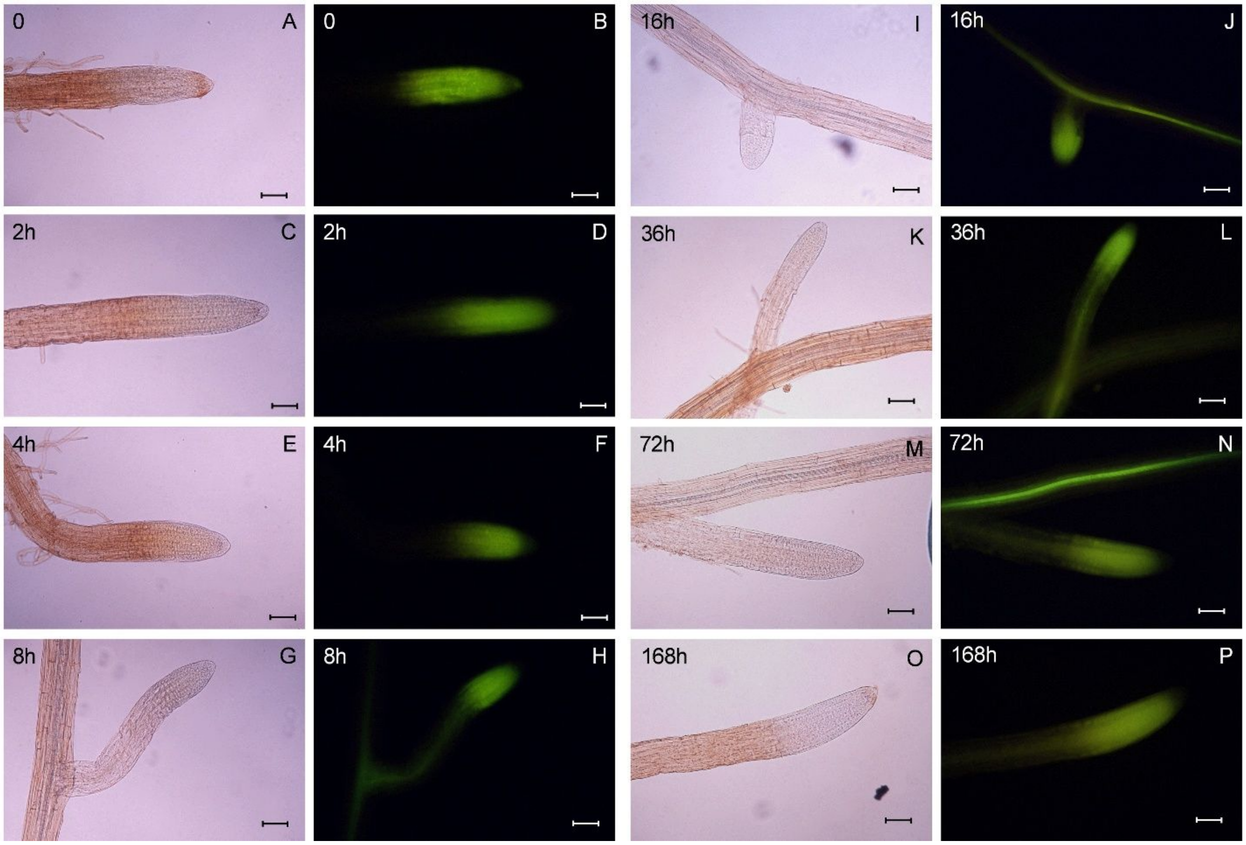

Figure S16. Representative bright-field (A, C, E, G, I, K, M, O) and corresponding epifluorescence (B, D, F, H, J, L, N, P) micrographs of Arabidopsis roots acquired at various time points over one week following incubation. During the first 4 h post-incubation, CDs-NH_2 fluorescence is localized near the meristematic apex, primarily within the elongation zone (A–F). Subsequently,

the signal extends into the vascular cylinder (G–P). The persistent fluorescence observed after 7 days (O–P) indicates that CDs-NH_2 remain stable within root tissues throughout the observation period. Scale bars: 50 μm.

Antioxidant activity of CDs-NH_2. The overall antioxidant activity of CDs-NH_2 was evaluated using both DPPH and ABTS free radicals as radical sources. DPPH is a stable free radical that, in its native form, displays a deep violet colour with a characteristic absorbance maximum at 520 nm; upon reaction with a reducing agent, it is converted into its yellow reduced form. ABTS, instead, can be readily oxidized in the presence of $(NH_4)_2S_2O_8$ to generate its radical cation ($ABTS^{\cdot +}$), which exhibits a strong absorbance maximum at 734 nm and readily reacts with a wide range of antioxidant species. Consequently, the decrease in absorbance at these wavelengths can be monitored to evaluate the antioxidant activity of the samples, as lower absorbance values correspond to higher radical-scavenging capacity.

According to literature, both radicals can be scavenged through either hydrogen-atom transfer (HAT) or electron transfer (ET) mechanisms[5-8], which are illustrated in **Figures S17** and **S18**, respectively.

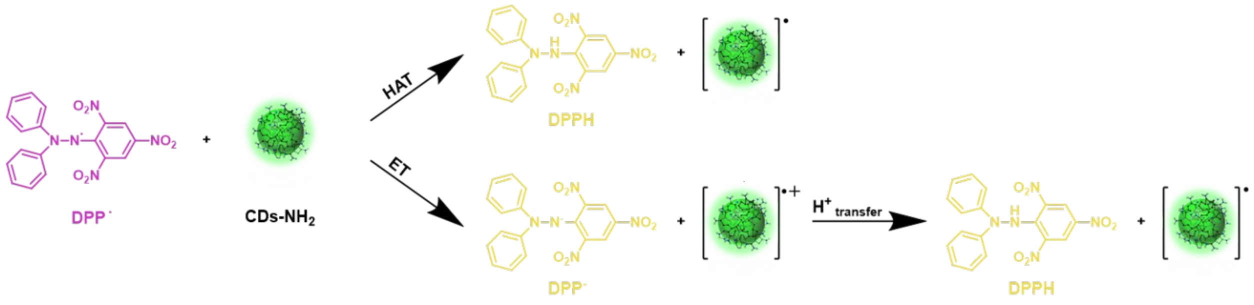

Figure S17. Mechanistic pathways of CDs-NH_2 scavenging DPPH radical via HAT and ET.

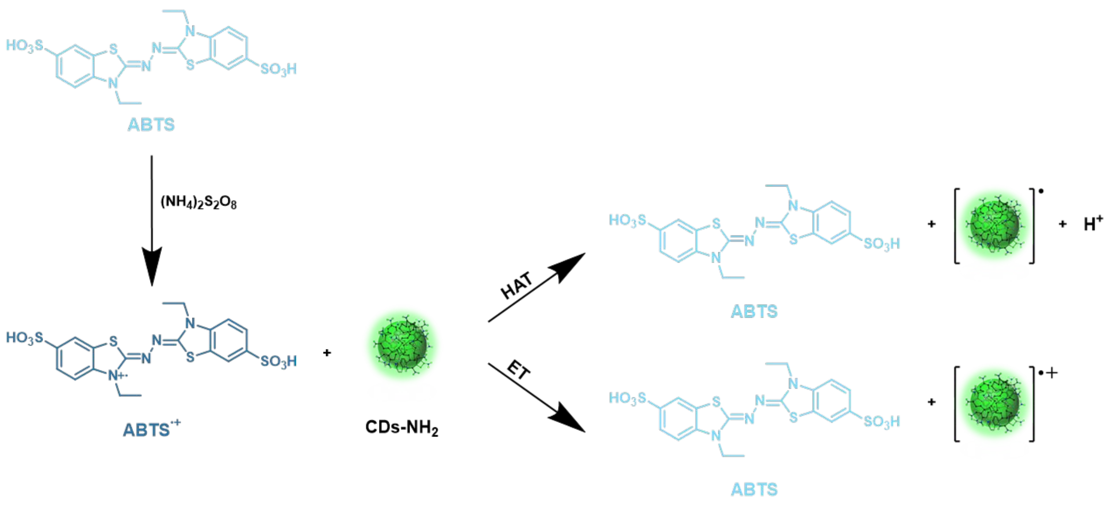

Figure S18. Generation of the ABTS radical cation and mechanistic pathways of CDs-NH_2 scavenging ABTS radical via HAT and ET.

Figure S19 shows photographs of the 96-well plate containing CDs–NH_2 used for the DPPH (A–B) and ABTS (C–D) assays, illustrating the colorimetric response recorded at time zero and after 60 minutes of reaction. **Figures S20A** and **B** show the radical scavenging activity of CDs-NH_2 against DPPH and ABTS, respectively. Both radicals exhibit a concentration-dependent response, with IC_{50} values of 62.5 µg/mL at time zero and 31.2 µg/mL after 30 and 60 minutes. For the ABTS assay, the samples at time zero displayed large error bars, which were attributed to incomplete solubilization of the ABTS solution; this issue disappeared in the subsequent measurements as the solution became fully solubilized.

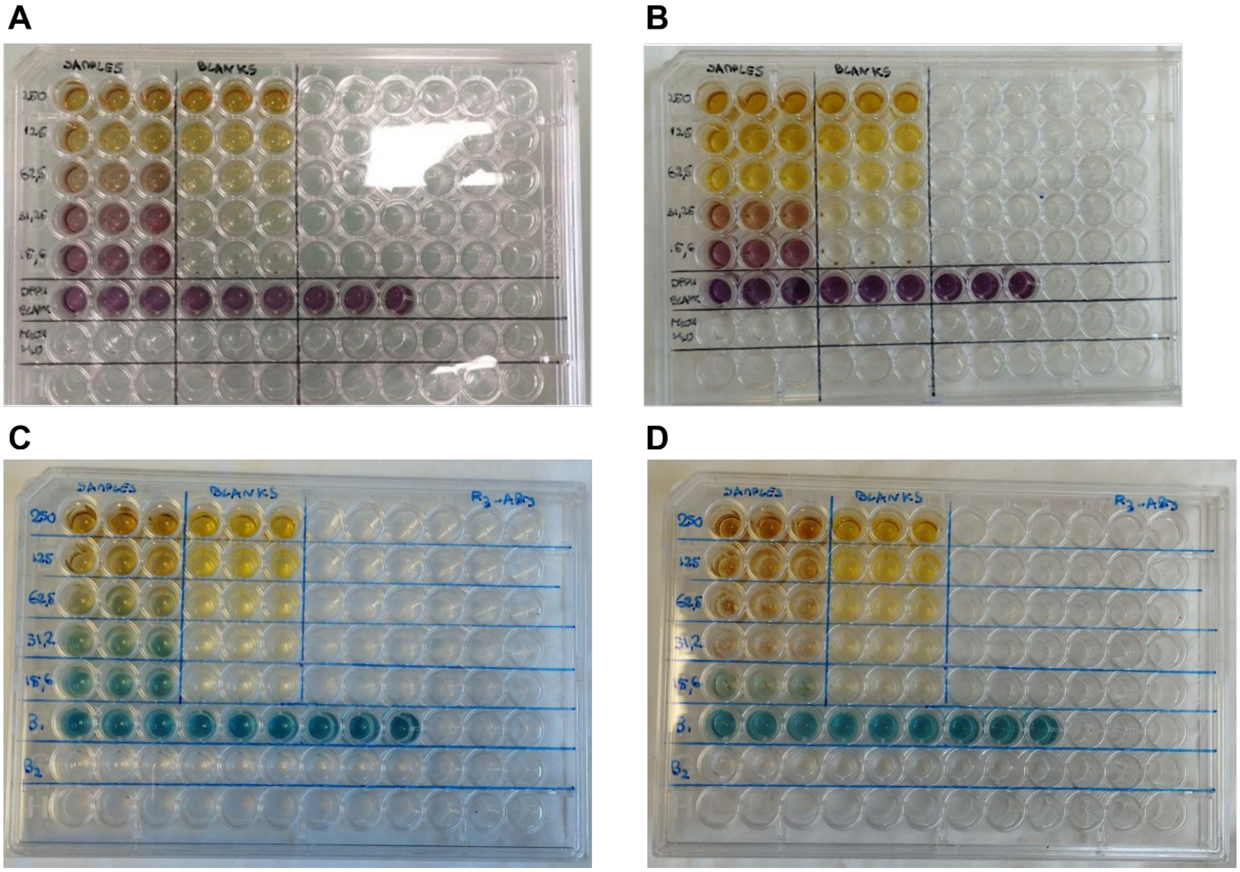

Figure S19. Photographs of the 96-well plates, where each horizontal row contains the CDs–NH_2 samples and the negative controls (NC) at decreasing concentrations (from top to bottom). The sixth row represents the positive control (PC), and the seventh row the solvent blank (SB). (A) and (B) show the DPPH plate before and after the treatment, respectively, while (C) and (D) show the ABTS plate before and after the treatment.

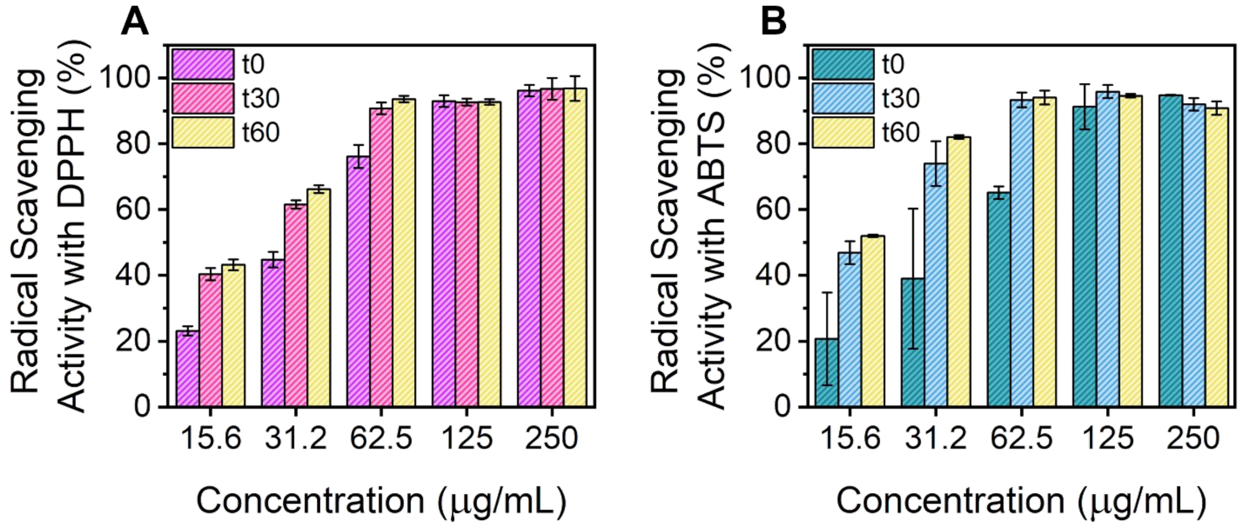

Figure S20. Radical scavenging activity of CDs-NH_2 at different time points (0, 30, and 60 minutes) against (A) DPPH and (B) ABTS. Each data represents the average of three independent measurements ± SE.

Bacterial growth inhibition. Figure S21 shows the inhibitory effect of CDs–NH_2 on *P. syringae* growth, highlighting a clear dose-dependent variation of the MIC with concentration.

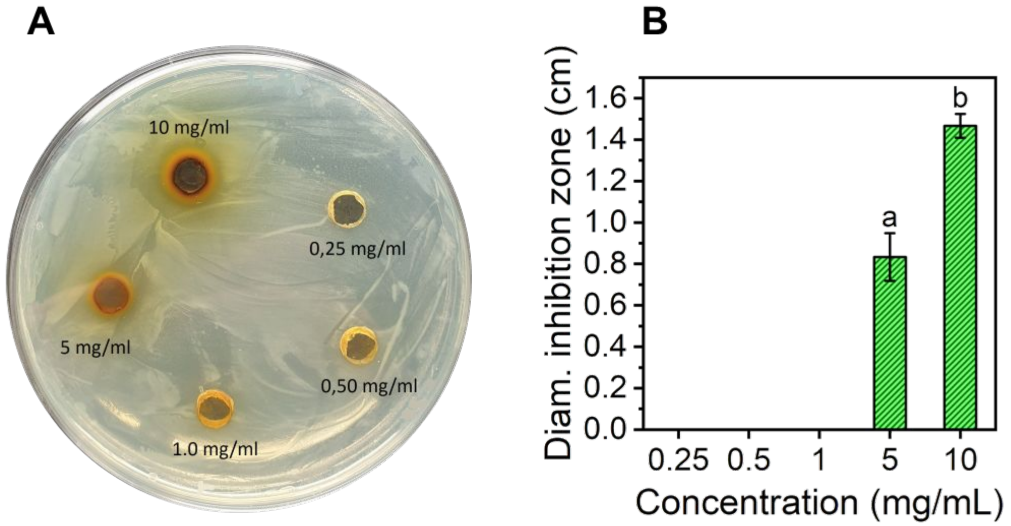

Figure S21. (A) Growth response of *P. syringae* pv. *tomato* (PtoDC3000) to different concentrations of CDs-NH_2. (B) Minimum inhibitory concentration (MIC); error bars indicate SE.

^{1}H NMR-based metabolomics analysis.

Table S2. Metabolites, resonance assignment, multiplicity and types of protons found in hydrophilic and lipophilic extracts of tomato leaves ^{1}H NMR spectrum. In bold are evidenced the resonances chosen for metabolite quantification; s: singlet, bs: broad singlet, d: doublet, t: triplet, q: quadruplet, dd: doublet of doublets, m: multiplet. Non-bold metabolites are ones observed in the spectra but non quantified due to signal overlapping or low sensitivity.

Compound	^{1}H δ ppm	Multiplicity	Assignment
**Amino acids**			
**Alanine**	**1.48**	**d**	**β-CH_3**
	3.80	q	α-CH
**Asparagine**	2.86	dd	β'-CH
	**2.89**	**dd**	**β-CH**
	4.01	m	α-CH
**Aspartic Acid**	2.70	dd	β'-CH
	**2.83**	**dd**	**β-CH**
	3.87	m	α-CH
**γ-Aminobutyric acid (GABA)**	**3.01**	**t**	**□-CH_2**
	2.28	t	α-CH_2
	1.89	m	β-CH_2
**Glutamic acid**	2.04	m	□-CH_2

	**2.36**	**m**	**β-CH_2**
	3.74	m	α-CH
**Glutamine**	**2.14**	**m**	**□-CH_2**
	2.45	m	β-CH_2
	3.81	m	α-CH
**Isoleucine**	0.95	t	-CH_3
	**1.02**	**d**	**g-CH_3**
	1.25	m	g’-CH
	1.49	m	g’’-CH
	1.99	m	β-CH
	3.69	m	α-CH
**Leucine**	**0.97**	**m**	**δ, δ’-CH_3**
	1.72	m	g-CH
	1.73	m	β-CH_2
	3.74	m	α-CH
**Phenylalanine**	**7.32**	**d**	**CH-2,6**
	7.38	d	CH-4
	7.42	d	CH-3,5
	3.27	m	β-CH_2
	3.98	dd	α-CH
**Threonine**	**1.32**	**d**	**g-CH_3**
	3.60	m	α-CH
	4.27	m	β-CH
**Tryptophan**	7.20	t	CH-5
	7.27	t	CH-6
	**7.53**	**d**	**CH-7**
	7.73	d	CH-4

**Tyrosine**	7.17<br>**6.89**<br>3.15<br>3.93	d<br>**d**<br>dd<br>dd	CH-2,6<br>**CH-3,5**<br>β-CH_2<br>α-CH
**Valine**	0.99<br>**1.05**<br>2.29<br>3.62	d<br>**d**<br>m<br>m	g-CH_3<br>**g’-CH_3**<br>β-CH<br>α-CH
**Organic Acids**			
**Ascorbic Acid**	**4.99**<br>3.77<br>3.72	**d**<br>td<br>d	**1-CH**<br>2-CH<br>CH_2
**Acetic Acid**	**1.92**	**s**	**CH_3**
**Citric Acid**	**2.69**<br>2.54	**d**<br>d	**[illegible]-CH_2**<br>α-CH_2
**Chlorogenic acid**	7.20<br>6.95<br>7.09<br>7.60<br>**6.37**	d<br>d<br>dd<br>d<br>**d**	CH-2’<br>CH-5’<br>CH-6’<br>CH-7’<br>**CH-8’**
**Formic Acid**	**8.46**	**s**	**CH**
**Fumaric Acid**	**6.51**	**s**	**CH=CH**
**Malic Acid**	**4.28**	**dd**	**α-CH**

	2.38; 2.69	dd	β, β'-CH
**Malonic Acid**	**3.11**	**s**	**CH_2**
**Neochlorogenic acid**	7.21<br>6.97<br>7.14<br>7.66<br>**6.43**	d<br>d<br>dd<br>d<br>**d**	CH-2'<br>CH-5'<br>CH-6'<br>CH-7'<br>**CH-8'**
**Quinic acid**	**2.04; 1.97**<br>2.08; 1.88<br>4.16<br>3.56<br>4.03	**dd**<br>dd<br>m<br>m<br>m	**2,2′-CH_2**<br>6,6′-CH_2<br>CH-3<br>CH-4<br>CH-5
**Succinic Acid**	**2,42**	**s**	**α, β-CH_2**
**U02 (Caffeoyl malic acid)**	**6.47**<br>7.69	**d**<br>d	**CH-8'**<br>CH-7'
**Carbohydrates**			
**Total Fructose (Fructofuranose+Fructopyranose)**	3.60-3.71<br>4.11<br>**3.84**<br>3.81	m<br>m<br>**m**<br>m	CH_2-1,1′<br>CH-3<br>**CH-5**<br>CH_2-6,6′
**α-Glucose**	**5.23**<br>3.55<br>3.72	**d**<br>m<br>m	**CH-1**<br>CH-2<br>CH-3

	3.42	m	CH-4
	3.84	m	CH-5
	3.73,3.90	m	CH_2-6
**β-Glucose**	**4.69**	**d**	**CH-1**
	3.26	m	CH-2
	3.50	m	CH-3
	3.42	m	CH-4
	3.48	m	CH-5
	3.74, 3.91	m	CH_2-6
**Oligosaccharides (Raffinose)**	5.45	d	GLC CH-1
	**5.01**	**d**	**GAL CH-1**
	4.22	d	FRU CH-3
**Sucrose**	**5.44**	**d**	**G CH-1**
	3.59	m	CH-2
	3.79	m	CH-3
	3.48	m	CH-4
	3.85	m	CH-5
	3.82	m	CH_2-6
	3.69	m	F CH_2-1’
	\	\	C-2
	4.22	m	CH-3’
	4.06	m	CH-4’
	3.90	m	CH-5’
	3.82	m	CH_2-6

**U01 ((1,4)-α-D-galacturonate), Polygalacturonic acid monomer)**	**5.06**<br>4.38<br>3.99<br>3.76	**d**<br>m<br>m<br>m	**CH-5'**<br>CH-4', CH-1'<br>CH-2'<br>CH-3'
**Lipids & Sterols**			
**Saturated fatty acid (SFA)**	0.87<br>1.26<br>1.62<br>**2.30**	t<br>m<br>m<br>**t**	CH_3<br>n-CH_2<br>C<u>H</u>$_2$-CH_2-CO_2^-<br>**CH_2-CO_2^-**
**Monounsaturated ω-9 fatty acid (ω-9 FA)**	0.88<br>1.27<br>**2.03**	t<br>m<br>**m**	CH_3<br>n-CH_2<br>**CH_2-CH=CH**
**Polyunsaturated ω-6 fatty acid (ω-6 FA)**	0.86<br>1.36<br>2.04<br>5.37<br>**2.76**<br>2.06<br>2.31	t<br>m<br>m<br>m<br>**t**<br>m<br>t	CH_3<br>n-CH_2<br>C<u>H</u>$_2$-CH=CH<br>CH=CH<br>**=CH-C<u>H</u>$_2$-CH=**<br>C<u>H</u>$_2$-CH_2-CO_2^-<br>CH_2-CO_2^-
**Polyunsaturated ω-3 fatty acid (ω-3 FA)**	0.95<br>1.37<br>2.04<br>5.36<br>**2.82**	t<br>m<br>m<br>m<br>**t**	CH_3<br>n-CH_2<br>C<u>H</u>$_2$-CH=CH<br>CH=CH<br>**=CH-C<u>H</u>$_2$-CH=**

	2.03	m	$C\underline{H}_2$-CH_2-CO_2^-
	2.30	t	CH_2-CO_2^-
**b-Sitosterol**	1.08, 1.85	m	CH_2-1
	1.51, 1.84	m	CH_2-2
	3.52	m	CHOH-3
	2.28	m	CH_2-4
	5.34	m	CH-6
	1.52, 1.98	m	CH_2-7
	1.46	m	CH-8
	0.99	m	CH-14
	1.57	m	CH_2-15
	1.26, 1.85	m	CH_2-16
	**0.68**	**s**	**CH_3-18**
	1.01	s	CH_3-25
**Campsterol (Camp)**	1.08, 1.85	m	CH_2-1
	1.51, 1.84	m	CH_2-2
	3.52	m	CHOH-3
	2.28	m	CH_2-4
	5.34	m	CH-6
	1.52, 1.98	m	CH_2-7
	1.46	m	CH-8
	0.99	m	CH-14
	1.57	m	CH_2-15
	1.26, 1.85	m	CH_2-16
	**0.70**	**s**	**CH_3-18**
	1.01	s	CH_3-25

**Other Metabolites**			
**Allantoin**	**5.38**	**s**	**CH**
***chiro*-Inositol**	4.05<br>3.76<br>**3.60**	m<br>m<br>**m**	CH-1,6<br>CH-2,5<br>**CH-3,4**
**Choline**	**3.21**<br>3.51<br>4.07	**s**<br>t<br>t	**$N\text{-}(CH_3)_3$**<br>CH_2<br>CH_2
**Dihydroxyacetone**	**4.41**	**s**	**CH_2, CH_2**
**Ethanolamine**	**3,13**<br>3.81	**t**<br>t	**CH_2-1**<br>CH_2-2
**Glycerophospholipids**	3.65-3,55<br>4.05-4.15<br>**5.21**	dd<br>dd<br>**m**	CH_2<br>CH_2<br>**CH**
***myo*-Inositol**	**3.54**<br>4.08<br>3.63<br>3.30	**m**<br>m<br>m<br>m	**CH-2,5**<br>CH-1<br>CH-3,6<br>CH-4
**Pheophitin a**	**9.53**<br>9.40	**s**<br>s	**CH-5**<br>CH-10
**Pheophitin b**	**11.2**<br>10.4	**s**<br>s	**CHO-7**<br>CH-10

**Total Carotenoids**	1.47<br>1.62<br>2.02<br>6.15<br>6.14<br>6.14<br>**6.68**<br>6.35<br>6.25<br>6.63<br>1.03<br>1.72<br>1.97	m<br>m<br>m<br>d<br>d<br>d<br>**m**<br>d<br>d<br>m<br>s<br>s<br>s	CH_2-2,2'<br>CH_2-3,3'<br>CH_2-4,4'<br>CH-7,7'<br>CH-8,8'<br>CH-10,10'<br>**CH-11,11'**<br>CH-12,12'<br>CH-14,14'<br>CH-15,15'<br>CH_3-16,16',17,17'<br>CH_3-18,18'<br>CH_3-19,19'
**Trigonelline**	**9.11**<br>8.83<br>8.11<br>8.83<br>4.43	**s**<br>m<br>m<br>m<br>s	**CH-1**<br>CH-3<br>CH-4<br>CH-5<br>CH_3
**Uridine moiety**	**7.86**<br>5.89	**d**<br>d	**1-CH**<br>2-CH
**2,3 Butanediol**	3.71<br>3.60<br>**1.13**	m<br>m<br>**d**	2-CH<br>3-CH<br>**1,4-CH_3**

Table S3. Quantification of each metabolite from the ^{1}H spectrum and significance with the ANOVA test. The significant variables of the ANOVA test were indicated with * for the significant differences in one way-ANOVA against controls (TL H_2O) with p<0.05, and with ** for the significant differences in one way-ANOVA against controls (TL H_2O) with p<0.01. Multiple groups comparison was corrected with the Dunnett test.

Metabolites	Amount (mg/100g)			
	TL H_2O	TL CDs-NH_2	TL Pto	TL CDs-NH_2+Pto
**Amino acids**				
Alanine	2.58 ± 0.72	4.09 ± 0.25	4.83 ± 1.24	4.33 ± 1.52
Asparagine	1.52 ± 0.13	2.77 ± 0.7	12.74 ± 11.93	11.95 ± 6.43
Aspartic acid	5.46 ± 0.64	7.35 ± 2.72	17.23 ± 3.33**	13.07 ± 1.83*
Isoleucine	0.24 ± 0.06	0.25 ± 0.05	1.35 ± 0.55*	1.01 ± 0.35
GABA	3.94 ± 0.91	1.61 ± 0.08	6.23 ± 3.19	2.96 ± 2.79
Glutamic acid	20.29 ± 0.97	20.31 ± 2.79	28.23 ± 4.7*	26.62 ± 1.53
Glutamine	13.58 ± 0.78	13.85 ± 1.64	24.38 ± 5.88*	20.36 ± 2.84
Glycine	7.86 ± 1.57	9.95 ± 0.82	17.35 ± 3.65*	14.42 ± 2.61
Leucine	0.24 ± 0.03	0.16 ± 0.02	1.82 ± 0.79*	1.15 ± 0.48
Threonine	1.61 ± 0.15	2.16 ± 0.42	2.41 ± 0.90	1.53 ± 0.29
Tryptophan	0.96 ± 0.07	0.69 ± 0.34	1.42 ± 0.36	1.46 ± 0.22
Tyrosine	0.68 ± 0.06	0.67 ± 0.12	1.14 ± 0.43	0.8 ± 0.16
Valine	0.44 ± 0.06	0.49 ± 0.01	1.86 ± 0.77**	1.07 ± 0.33
**Carbohydrates and polyols**				

**Chiro-inositol**	52.7 ± 4.68	60.84 ± 0.47	64.15 ± 9.91	59.83 ± 4.40
**Glucose**	9.07 ± 1.9	10.57 ± 2.53	5.27 ± 1.87	4.42 ± 0.24*
**Sucrose**	39.85 ± 11.12	54.81 ± 4.38	94.13 ± 20.02*	76.25 ± 13.57
**Myo-inositol**	10.11 ± 1.23	9.92 ± 1.74	8.37 ± 1.22	8.05 ± 1.45
**Total fructose**	18.2 ± 2.43	20.05 ± 1.67	12.36 ± 3.66	13.25 ± 0.90
**U01 (Polygalacturonic acid)**	5.47 ± 1.61	4.73 ± 1.18	2.24 ± 1.28*	1.36 ± 1.14**
**Organic acids**				
**4-Hydroxybenzoic acid**	0.13 ± 0	0.17 ± 0.06	0.25 ± 0.14	0.15 ± 0.06
**Ascorbic acid**	0.12 ± 0.04	0.14 ± 0.03	0.16 ± 0.06	0.17 ± 0.02
**Chlorogenic acid**	2.5 ± 0.3	2.3 ± 0.67	1.04 ± 0.7*	0.41 ± 0.53**
**Citric acid**	166.06 ± 11.56	152.23 ± 37.63	124.33 ± 20.16	79.44 ± 47.29**
**Formic acid**	0.8 ± 0.36	1.07 ± 0.12	1.04 ± 0.26	1.02 ± 0.03
**Malic acid**	49.4 ± 6.96	51.19 ± 9.40	36.06 ± 3.95	20.86 ± 13.05*
**Malonic acid**	1.82 ± 0.28	2.61 ± 1.02	4.35 ± 2.79	5.08 ± 2.82
**Neochlorogenic acid**	5.48 ± 1.46	5.43 ± 1.48	2.63 ± 1.24	1.3 ± 1.15*
**Quinic acid**	0.97 ± 0.1	0.88 ± 0.1	1.07 ± 0.1	0.73 ± 0.21
**U02 (Caffeoyl malic acid)**	12.02 ± 2.85	11.03 ± 3.71	4.54 ± 2.7*	1.97 ± 2.71**
**Lipids and sterols**				
**β-Sitosterol**	3.14 ± 0.34	3.3 ± 0.79	3.86 ± 0.84	3.33 ± 1.37
**Camposterol**	0.9 ± 0.09	0.91 ± 0.14	1.19 ± 0.11	1 ± 0.26
**Omega 3 FA**	61.31 ± 11.51	53.02 ± 6.06	46.87 ± 9.49	33.16 ± 10.51*
**Omega 6 FA**	1.29 ± 0.25	1.22 ± 0.3	2.35 ± 0.65	2.47 ± 0.74

**Omega 9 FA**	20.74 ± 2.38	15.39 ± 1	17.06 ± 1.55	16.45 ± 6.9
**Total saturated fatty acids**	38.69 ± 5.85	46.4 ± 3.81	59.05 ± 17.09	45.42 ± 14.2
**Total unsaturated fatty acids**	84.23 ± 12.17	70.41 ± 5.46	66.98 ± 11.4	52.57 ± 17.37*
**Other compounds**				
**2,3-Butanediol**	0.19 ± 0.02	0.15 ± 0.03	0.19 ± 0.03	0.23 ± 0.05
**Allantoin**	5.47 ± 0.94	6.76 ± 1.28	7.92 ± 1.26	8.73 ± 1.28*
**Betaine**	0.72 ± 0.11	0.87 ± 0.1	0.83 ± 0.48	0.39 ± 0.3
**Choline**	7.24 ± 0.54	8.28 ± 0.53	8.12 ± 1.62	7.53 ± 0.93
**Dihydroxyacetone**	4.4 ± 0.14	4.7 ± 0.44	5.35 ± 1.57	5.11 ± 1.36
**Ethanolamine**	2.06 ± 0.16	2.32 ± 0.19	2.23 ± 0.51	1.76 ± 0.18
**Glycerophospholipids**	1.63 ± 0.42	1.13 ± 0.3	2.55 ± 0.71	1.87 ± 0.71
**Phaeophytin A**	16.64 ± 2.93	20 ± 1.93	17.32 ± 4.68	9.97 ± 6.81
**Phaeophytin B**	3.78 ± 0.59	4.44 ± 0.27	3.89 ± 2	2.96 ± 1.04
**Total carotenoid**	77.33 ± 11.22	63.83 ± 3.07	69.55 ± 12.4	54.62 ± 13.75
**Trigonelline**	4.56 ± 0.26	4.94 ± 0.53	5.6 ± 1.62	5.26 ± 1.35
**Uridine moiety**	2.8 ± 0.08	2.68 ± 0.25	3.13 ± 0.56	3.36 ± 0.63

Metabolomics multivariate analysis.

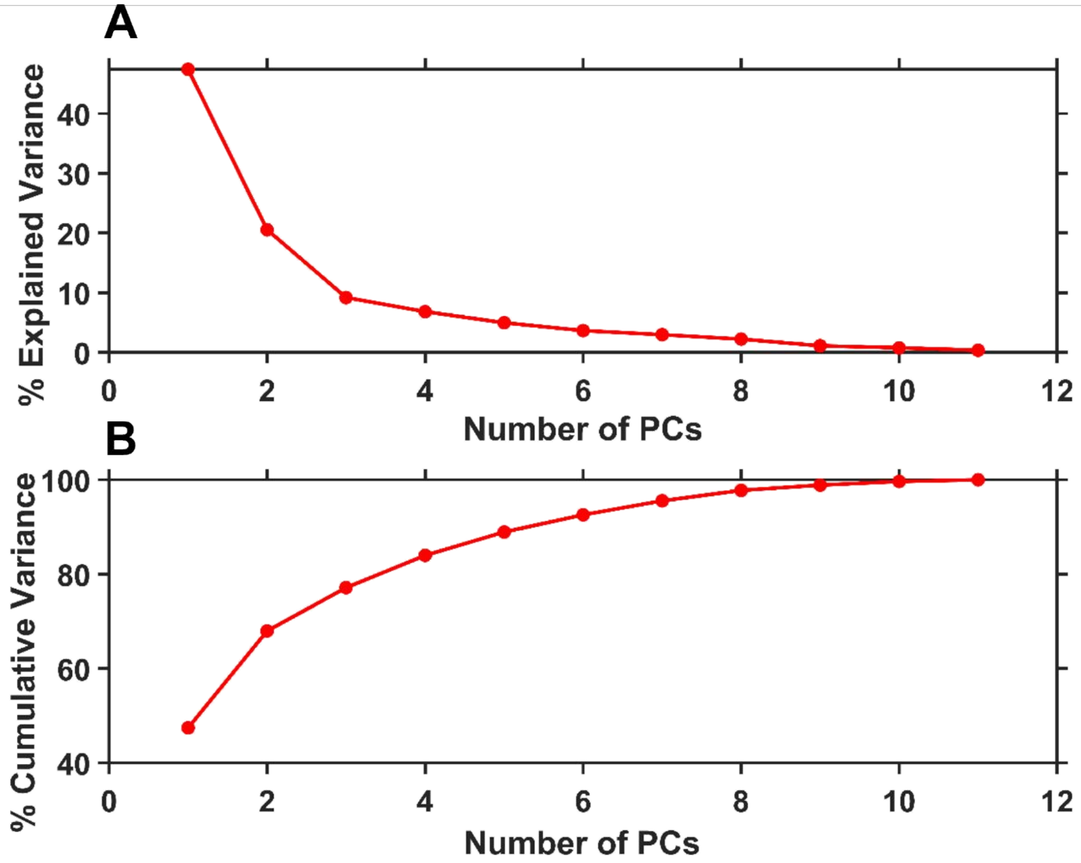

Figure S22. PCA (A) Cumulative and (B) explained variance plot. The first plot shows the percentage of variance from the original data matrix retained by each Principal Component (PC) of the PCA, while the second plot shows the Cumulative percentage of variance per PCs. For the Tomato leaves (TL) dataset, the sample's separation according to the experimental design is present on the first PC, retaining 47.4% of variance from the original data matrix.

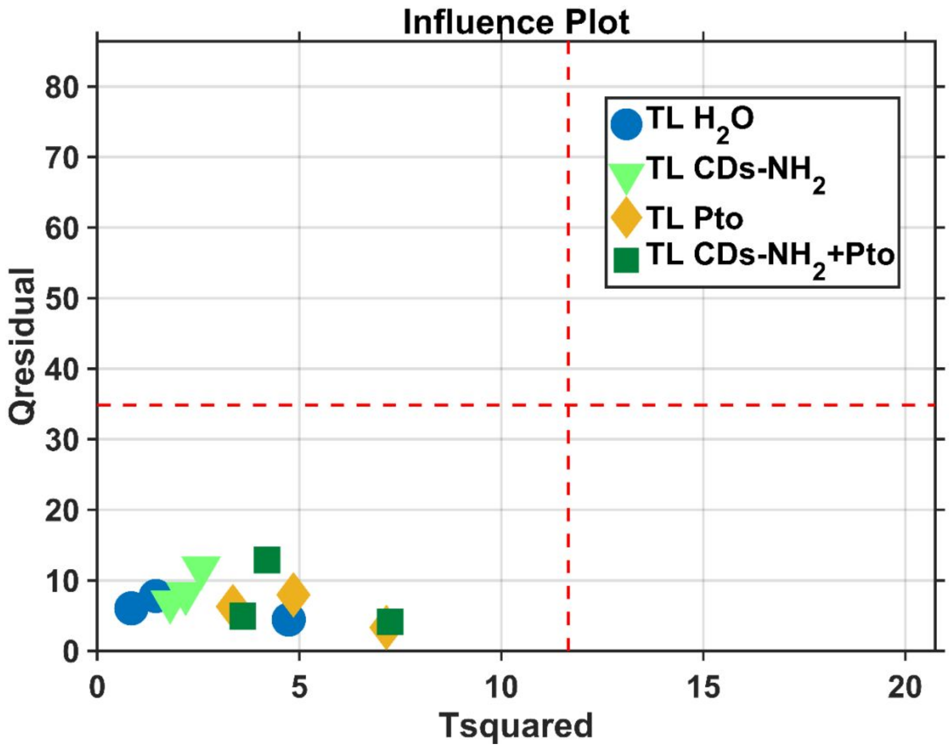

Figure S23. Influence plot of tomato leaf samples, H_2O, CDs-NH_2, Pto, CDs-NH_2+Pto, used for outlier detection. Tsquared is calculated using Mahalanobis distance in the PCA space, as a measure of distance for each sample from the centre of the multivariate space. Qresidual is calculated from the residuals of the original data matrix and the reconstructed one from the PCA, retaining the chosen Principal Components (PCs). In red are shown 95% confidence intervals of both Tsquared and Qresidual values.

REFERNCES

1. Sturabotti, E.; Camilli, A.; Moldoveanu, V. G.; Bonincontro, G.; Simonetti, G.; Valletta, A.; Serangeli, I.; Miranda, E.; Amato, F.; Marrani, A. G.; Migneco, L. M.; Sennato, S.; Simonis, B.; Vetica, F.; Leonelli, F., Targeting the Antifungal Activity of Carbon Dots against Candida albicans Biofilm Formation by Tailoring Their Surface Functional Groups. *Chem. Eur. J.* **2024**, *30*, e202303631.

2. Tuinstra, F.; Koenig, J. L., Raman Spectrum of Graphite. *J. Chem. Phys.* **1970,** *53* (3), 1126-1130.

3. Mallet-Ladeira, P.; Puech, P.; Toulouse, C.; Cazayous, M.; Ratel-Ramond, N.; Weisbecker, P.; Vignoles, G. L.; Monthioux, M., A Raman study to obtain crystallite size of carbon materials: A better alternative to the Tuinstra–Koenig law. *Carbon* **2014,** *80*, 629-639.

4. Cuesta, A.; Dhamelincourt, P.; Laureyns, J.; Martínez-Alonso, A.; M. D. Tascón, J., Comparative performance of X-ray diffraction and Raman microprobe techniques for the study of carbon materials. *J. Mater. Chem.* **1998,** *8* (12), 2875-2879.

5. Gulcin, İ.; Alwasel, S. H. DPPH Radical Scavenging Assay. *Processes* **2023**, *11* (8), 2248.

6. Innocenzi, P.; Stagi, L. Carbon Dots as Oxidant-Antioxidant Nanomaterials: Understanding the Structure-Properties Relationship. *Nano Today* **2023**, *50*, 101837.

7. Apak, R.; Özyürek, M.; Güçlü, K.; Çapanoğlu, E. Antioxidant Activity/Capacity Measurement. 1. Classification, Physicochemical Principles, Mechanisms, and Electron Transfer (ET)-Based Assays. *J. Agric. Food Chem.* **2016**, *64* (5), 997–1027.

8. Apak, R.; Özyürek, M.; Güçlü, K.; Çapanoğlu, E. Antioxidant Activity/Capacity Measurement. 2. Hydrogen Atom Transfer (HAT)-Based, Mixed-Mode (Electron Transfer (ET)/HAT), and Lipid Peroxidation Assays. *J. Agric. Food Chem.* **2016**, *64* (5), 1028–1045.
